# Isoquercitrin mitigates intestinal ischemia-reperfusion injury by regulating intestinal flora and inhibiting NLRP3 inflammasome activation

**DOI:** 10.1016/j.redox.2025.103803

**Published:** 2025-08-05

**Authors:** Hui Xu, Tian-qi He, Su-ying Chen, Rui-rui Shi, Jian Xu, Yu-run Xing, Dan Shi, Yi-qin Liu, Bo-sheng He, Jin-hua Gu

**Affiliations:** a Nantong Institute of Genetics and Reproductive Medicine, Department of Pharmacy, Affiliated Maternity and Child Healthcare Hospital of Nantong University, Nantong, China; b School of Pharmacy, Nantong University, Nantong, China; c Department of Pharmacy, Taixing People's Hospital, Taizhou, China; d Department of Radiology, Affiliated Hospital 2 of Nantong University, Nantong, China

**Keywords:** Isoquercitrin, Intestinal ischemia-reperfusion, Intestinal flora, Nrf2/HO-1, Inflammatory cytokines

## Abstract

Intestinal ischemia-reperfusion (II/R) injury, frequently observed in clinical emergencies such as trauma, infection, and transplantation, leads to severe epithelial necrosis, loss of villi, and alarmingly high mortality rates (50 %–90 %), yet current pharmaceutical treatments largely prove ineffective. This study employs network pharmacology alongside *in vivo* and *in vitro* experiments to explore the potential of isoquercitrin, a flavonoid abundant in various dietary sources and known for its anti-inflammatory and antioxidant properties, in mitigating intestinal II/R injury. We found that isoquercitrin significantly reinforced the integrity of the intestinal barrier and markedly alleviated damage associated with II/R injury. Additionally, it enhanced the intestinal microbiota structure by promoting microbial diversity and supporting beneficial bacterial populations. According to network pharmacology analyses, isoquercitrin may prevent II/R injury by modulating redox-related pathways and regulating inflammatory responses mediated by the NLRP3 inflammasome. This protective effect is evidenced by reduced levels of reactive oxygen species (ROS) and malondialdehyde (MDA), as well as an increased GSH/GSSG ratio and enhanced superoxide dismutase (SOD) activity. Isoquercitrin also inhibited NLRP3 inflammasome activation and decreased the expression of downstream factors, including Caspase-1, IL-1β, IL-6, and keratinocyte-derived cytokine (KC). The observed effects correlate with enhancement of nuclear translocation of nuclear factor erythroid 2-related factor 2 (Nrf2) and increased expression of heme oxygenase-1 (HO-1) in a dose-dependent manner, and these beneficial effects were abolished by both ML385 (an Nrf2 inhibitor) and siNrf2. Thus, activating the Nrf2/HO-1 signaling pathway is crucial to isoquercitrin's protective role in intestinal II/R injury. The present findings underscore the therapeutic potential of isoquercitrin in managing intestinal II/R injury.

## Highlights

• Isoquercitrin (IQ) effectively reduces intestinal ischemia/reperfusion injury.
• IQ fortifies the intestinal barrier and reduces epithelial necrosis and villi loss.
• IQ increases microbial diversity and fosters beneficial bacteria in the gut.
• IQ suppresses NLRP3-mediated inflammation.
• IQ mitigates oxidative stress and restores intestinal barrier functions via the Nrf2/HO-1 pathway.

## Graphical abstract

The protective effect of isoquercitrin against II/R injury in mice.

**Figure:**
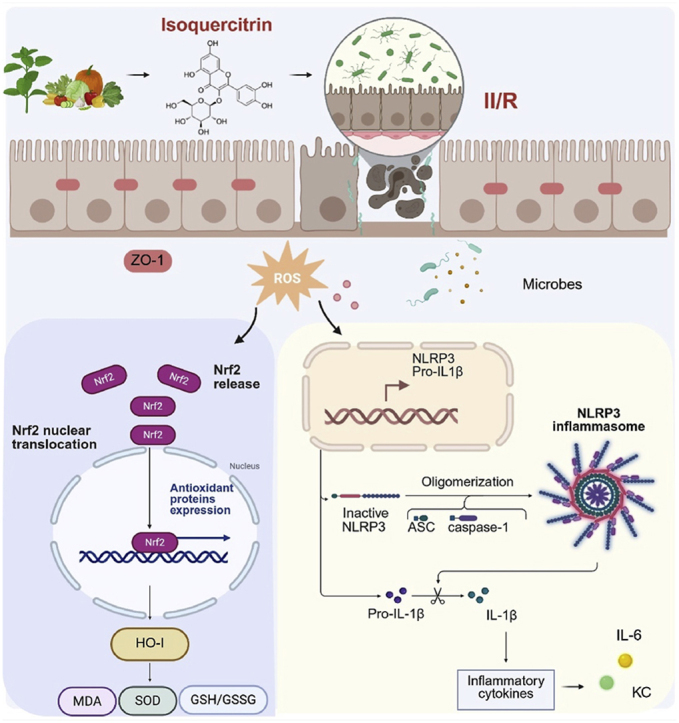

## 1 Introduction

The intestinal ischemia-reperfusion (II/R) injury is a frequent complication in a variety of clinical scenarios, such as trauma, hemorrhagic shock, intestinal obstruction, and acute mesenteric ischemia [1]. The ischemia following the blood flow disruption induces epithelial damage to the intestine [2], and the subsequent reperfusion, while reoxygenating the tissue, exacerbates the injury. The II/R injury is associated with many disorders, including necrotizing enterocolitis, graft rejection in small intestine transplants, complications after abdominal aortic aneurysm surgery, and inflammatory bowel disease [3]. Acute mesenteric ischemia in particular poses a life-threatening emergency and usually results in severe morbidity and high mortality [4,5]. Researchers have investigated the pathophysiology of the II/R injury and developed various therapeutic interventions, such as anti-inflammatory drugs, apoptosis inhibitors, antioxidants, and immune response modulators. For instance, ginsenosides can inhibit NF-κB to ameliorate the II/R injury [6], lycopene mitigates the inflammatory response triggered by II/R [7], and serotonin contributes to the restoration of the intestinal mucosa [8].

As the main active ingredients of many traditional Chinese medicines, flavonoids have been used to improve cardiovascular function, prevent and treat hypertension, address complications associated with metabolic abnormalities, and manage various other clinical scenarios [[9], [10], [11]]. Isoquercitrin (IQ), a monoglucoside of the flavonoid quercetin, is commonly found in herbs, vegetables, and plants and is safe, stable, inexpensive, and readily available [12]. It is widely used in food and medicine because of its multifaceted biological properties, which encompass antioxidant, anti-inflammatory, and anti-apoptotic effects [13]. It is used for the synthesis of enzyme-modified isoquercitrin (EMIQ), which has been approved by the FDA as a food additive [14]. Isoquercitrin can significantly ameliorate neurological deficits [15], suppress the secretion of pro-inflammatory cytokines [16], and reduce apoptosis [17]. It can mitigate oxidative stress and neuronal apoptosis to protect against ischemic stroke by suppressing the NOX4/ROS/NF-κB pathway [18]. However, the effectiveness of IQ in mitigating II/R injury remains to be further understood.

The intestinal mucosal barrier provides chemical, mechanical, immunological, and biological defenses, and its dysfunction allows intestinal microbes, endotoxins, and free radicals to enter the peripheral tissues via the bloodstream. The dysfunction of this barrier, which is a major manifestation of the II/R injury [[19], [20], [21]], can lead to systemic inflammatory response syndrome, multiple organ dysfunction syndrome, and in severe cases, death. Recent investigations have revealed that the gut microbiota and their metabolic products to some extent protect the intestinal and extraintestinal organs against damages caused by II/R injury. Probiotics such as *Bifidobacterium bifidum* PRL2010 can attenuate II/R injury, inhibit neutrophil infiltration, reduce oxidative stress, and limit the translocation of gut flora in distant organs like liver and kidneys [22]. Wang et al. reported that Bifidobacteria can suppress the elevated inflammatory mediators and moderate apoptosis in intestinal cells following II/R, thereby preserving mucosal integrity and junctional complexes and hindering microbial translocation [23]. Salim et al. reported that the long-term administration of the probiotic VSL#3 could alleviate small intestinal inflammation by downregulating immune cell recruitment and modulating the levels of pro-inflammatory cytokines [24]. Wang et al. disclosed that *Lactobacillus plantarum* could mitigate II/R injury by alleviating the apoptosis of the intestinal epithelial cells and preventing bacterial translocation [25]. Hu et al. observed a strong inverse relationship between the presence of *Lactobacillus murinus* in postoperative patients’ feces and the extent of their II/R injury [26]. These findings underscore the complex relationship between the II/R injury, intestinal mucosal barrier, and gut microbiome.

The regulation of reactive oxygen species (ROS) is critical in treating II/R injury, as these molecules not only activate pro-inflammatory cytokines but also participate in signaling cascades that regulate gene expression and cellular functions. Upon ischemia, ROS are quickly formed and accumulate in the intestinal tract, which disrupts the equilibrium between oxidant and antioxidant levels, and various oxidation factors are then generated and cause peroxidative damage by activating oxidative pathways. Nuclear factor erythroid 2-related factor 2 (Nrf2) is a crucial transcription factor that detects oxidative stress. It promotes the expression of genes encoding antioxidant, anti-inflammatory, and detoxifying proteins and thus plays a vital role in protecting cells from exogenous substances and oxidative damage [27]. Under normal conditions, Nrf2 is predominantly localized in the cytoplasm, where it is kept inactive through binding with the negative regulator Kelch-like ECH-associated protein 1 (Keap1). However, in response to oxidative stress or cellular damage, Nrf2 dissociates from Keap1 and translocates to the nucleus, where it binds to the antioxidant response element in the promoter regions of target genes. This nuclear translocation activates the transcription of cytoprotective genes, including phase II detoxifying enzymes such as heme oxygenase-1 (HO-1) and NAD(P)H quinone oxidoreductase 1 (NQO-1) [28], which in turn enhance antioxidant defenses and support cellular homeostasis [29].

The NLRP3 inflammasome serves as a sensor of microbial infection and cellular damage and is a key component of innate immunity. Upon activation, it promotes the maturation of Caspase-1, which in turn drives the release of proinflammatory cytokines IL-1β and IL-18 [30]. While this immune signaling is protective in acute infection, dysregulated NLRP3 activity has been implicated in chronic inflammatory conditions such as Alzheimer's disease, diabetes, atherosclerosis, etc. [31]. Its overactivation is also linked to inappropriate inflammatory responses against bacterial, fungal, and viral pathogens. Stress signals such as changing ion flux, mitochondrial dysfunction, ROS accumulation, and lysosomal rupture have been identified as triggers [32]. Researchers now agree that the Nrf2/HO-1 axis can influence NLRP3 activation by regulating intracellular ROS levels [33,34].

It has been found that IQ can inhibit NF-κB and caspase-3, resulting in reduced TNF-α, IL-1β, and IL-6 levels after ischemic stroke [18]. However, research is currently lacking on how IQ may ameliorate II/R injury. The present work thus seeks to bridge this knowledge gap by exploring how IQ mitigates II/R injury. This work created animal and cell models to systemically examine the effects of IQ on the intestinal barrier function, gut microbiota, oxidative stress, inflammation, and the Nrf2/HO-1 axis. The results provide a scientific foundation for clinical practices and may help to identify molecular targets and develop new treatments for II/R injury.

## 2 Materials and methods

### 2.1 Network pharmacology research

#### 2.1.1 Collection of IQ target sites

The 3D structure of IQ was obtained from the PubChem database, and corresponding targets were identified using the SwissTarget database. The target protein information was standardized using the Uniprot protein database.

#### 2.1.2 Collection and screening of II/R targets

Using “intestinal ischemia-reperfusion injury” as a keyword, we searched the GeneCards, OMIM, and DrugBank databases to compile information on disease targets.

#### 2.1.3 Construction of the protein interaction network for disease targets

The predicted targets obtained from the intersection of IQ and II/R data were imported into the STRING online platform, with the species set to “*Mus musculus*” and confidence level set at >0.4 (moderate confidence). This created a protein–protein interaction network for the disease targets. The data were then imported into Cytoscape 3.7.2, where BisoGenet and CytoNCA plugins were used to analyze drug and disease targets. Topological analysis based on Degree, Betweenness Centrality, and Closeness Centrality was conducted to identify the network's core targets, with node sizes set according to Degree.

#### 2.1.4 GO enrichment analysis and KEGG pathway enrichment analysis

Potential target information underwent GO and KEGG enrichment analyses using DAVID, with identifiers set to “OFFICIAL_GENE_SYMBOL” and the species to “*Mus musculus*”. The results were visualized using R [35].

#### 2.1.5 Molecular docking between IQ and target proteins

Molecular docking was performed using AutoDockTools-1.5.6, with binding energy as the criterion for assessing compound–target binding affinity, in accordance with the methodology described in previous studies [35,36]. The final visualization was conducted using Discovery Studio 2019.

### 2.2 Materials

IQ was extracted from *Apocynum venetum* Linn. (apocynaceae) and purchased from MedChemExpress (99.87 %; HY-N1445R; Monmouth Junction, NJ, USA). The chemical structure of IQ is shown in Fig. 5A. Salidroside was isolated and extracted from the dried root of *Rhodiola crenulata* and sourced from Herbpurify Co., Ltd (≥98 %, HPLC purity; 100338-51-9; Chengdu, China). ML385 was sourced from MedChemExpress (HY-100523). The stock solutions (in DMSO) of IQ (10 mg/mL), ML385 (50 mg/mL), or salidroside (20 mg/mL) were diluted with PBS to desirable levels before use.

### 2.3 Animal models of II/R injury

**Fig. 1:**
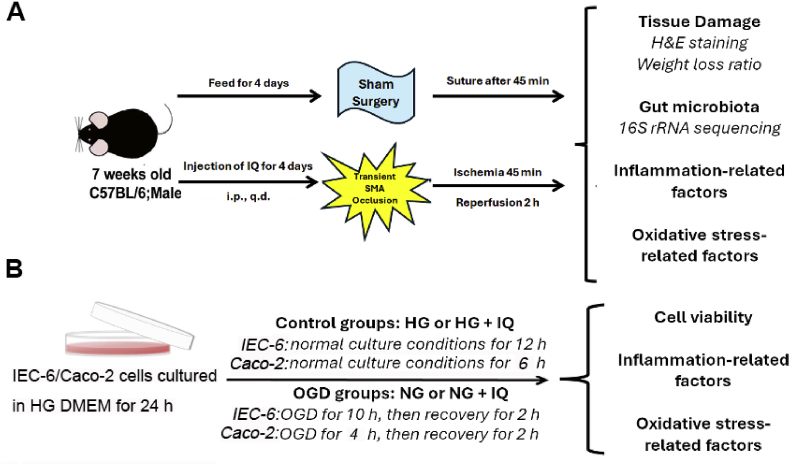
Model construction. (A) Mouse model with II/R injury. i.p., intraperitoneal; q.d., once a day; SMA, superior mesenteric artery. (B) Cell model with oxygen glucose deprivation and reperfusion (OGD/R). HG, high glucose; NG, no glucose.

Adult male C57BL/6 mice (6–8 weeks old, 25 ± 3 g) were obtained from Vital River (Beijing, China) and raised in specific pathogen-free environments, with unlimited food and water, under a 12 h/12 h diurnal cycle. The acclimation period was 3 days. The mice were randomly assigned to five groups of eight mice each (Fig. 1A) and received sham surgery (Group M1) or the II/R surgery (Groups M2–M5). Prior to the procedure, all mice were fasted for 10 h but had free access to water. IQ was administered intraperitoneally to the mice in Groups M3, M4, and M5 at 10, 20, and 40 mg/kg, respectively, once daily for four consecutive days leading up to surgery.

To begin the surgery, the mice were anesthetized with 2.5 % Avertin®. An incision (about 1 cm) was then made along the midline of the upper abdomen, and the abdominal fascia and the peritoneum were separated bilaterally to expose the abdominal cavity and the intestinal tissues. For mice in Groups M2–M5, the superior mesenteric artery (SMA) was carefully separated from the root of the mesentery, and the roots of the two ends of the SMA were ligated with nylon sutures. The operation decreased the mesenteric and intestinal blood flow, and intestinal ischemia was visually confirmed by tissue whitening. After ischemia for 45 min, the knots on the SMA were gently loosened to restore the arterial blood flow, and the incision was closed. For the mice in Group M1, the incision was closed after exposure of the abdominal cavity for 45 min.

Once the incision was closed, the mice were given 2 h of reperfusion, during which time they recovered in their cage and could drink water freely (but got no food). They were then euthanized by cervical dislocation. Blood was collected from the heart. The intestinal segment (2 cm) harvested from the fixation site was weighed immediately to give the wet weight (*w*_w_), and it was then baked at 80 °C for 4–6 h and weighed to give the dry weight (*w*_d_). The loss ratio was calculated as γ = (*w*_w_ − *w*_d_)/*w*_d_. The temperature was kept at 23–25 °C during the surgery and recovery.

All animal experiment procedures adhered to the National Institutes of Health guidelines for the use and care of animals and were approved by the Nantong University Animal Research Ethics Committee (Approval No. 20221211007).

### 2.4 Cell models of OGD/R

IEC-6, a rat small intestinal crypt epithelial cell line, was acquired from American Type Culture Collection (Manassas, VA, USA). The Caco-2 cell line was obtained from Shanghai Institutes for Biological Science (Shanghai, China). Cells were cultured at 37 °C in a humidified 5 % CO_2_ atmosphere using a high glucose Dulbecco's Modified Eagle Medium (DMEM; 4.5 g/L glucose; C11995500BT, Gibco, Grand Island, NY, USA). The cells were divided into separate groups (Fig. 1B) and underwent OGD/R and/or treatment with IQ (IEC-6: 6.25–100 μM; Caco-2: 25–400 μM) if applicable. Salidroside (25 μM) was used as the positive control.

To establish the OGD/R models, cells were incubated at 37 °C in glucose-free DMEM (0 g/L glucose; 11966-025, Gibco) containing IQ and/or ML385 as applicable. The plates were then placed in a portable anoxic tank maintained at 37 °C, which was purged with 95:5 v/v N_2_/CO_2_ for 5 min before the valve was closed. After hypoxia treatment (IEC-6: 10 h; Caco-2: 4 h), the plates were retrieved, and the medium was replaced with high-glucose DMEM (4.5 g/L) supplemented with IQ and/or ML385 as appropriate. The plates were then placed in the incubator and kept at 37 °C for an additional 2 h. The incubator provided a humidified environment with 5 % CO_2_. The control cells (i.e., without OGD/R) were incubated analogously but under air.

### 2.5 Characterizations

#### 2.5.1 Western blot

After the homogenized ischemic intestinal tissue was centrifuged, the supernatant was collected, and its protein concentration was measured using a BCA assay kit (P0011; Beyotime, Shanghai, China). The supernatant was diluted to an appropriate level according to the assay result, and 1 × loading buffer was added. The mixture was boiled at 95 °C for 5 min, cooled to room temperature, and stored at −20 °C.

Protein extracts were resolved by 10 %–12.5 % sodium dodecyl sulfate polyacrylamide gel electrophoresis (SDS-PAGE) and transferred onto PVDF membranes (Millipore, Bedford, MA, USA). The membranes were blocked for 1 h at room temperature in 5 % skimmed dry milk prepared in TBST, then incubated with the primary antibodies at 4 °C overnight and with the secondary antibodies at room temperature for 2 h, and visualized by electroluminescence (ECL) using an Odyssey Imaging System. The results were statistically analyzed using ImageJ. The primary antibodies included NLRP3 (30109-1-AP, 1:1000), GAPDH (10494-1-AP, 1:1000), IL-1β (26048-1-AP, 1:2000), Caspase-1 (22915-1-AP, 1:5000), Nrf2 (16396-1-AP, 1:1000), HO-1 (10701-1-AP, 1:2000), Lamin B1 (12987-1-AP, 1:1000), apoptosis-associated speck-like protein containing a CARD (ASC, 67824, 1:1000), and ZO-1 (21773-1-AP, 1:5000). The secondary antibodies were horseradish peroxidase-coupled goat anti-mouse IgG (SA00001-1, 1:5000) and horseradish peroxidase-coupled goat anti-rabbit IgG (SA00001-2, 1:5000). ASC was purchased from Cell Signaling Technology (Danvers, MA, USA), and all other antibodies were purchased from Proteintech (Rosemont, IL, USA).

#### 2.5.2 16S rRNA sequencing

The collected mouse intestinal contents were sent to Jiangsu Sanshu Biotechnology Co., Ltd. (Nantong, China) for 16S rRNA sequencing. The extracted genomic DNA was detected by 1 % agarose gel electrophoresis, and online sequencing used the library constructed after PCR amplification of the genomic DNA.

#### 2.5.3 Cell transfection with Nrf2 siRNA

Caco-2 cells (1 × 10^5^/well) were seeded in six-well plates. Upon reaching 50 %–70 % confluence, the cells were transfected with siNC and siNrf2 using *Lipofectamine*® *3000* (L3000015; Invitrogen, Carlsbad, CA, USA). The transfected cells were then subjected to OGD/R and treated with or without IQ.

#### 2.5.4 Cell viability

The CCK8 stock solution (C0038; Beyotime) was diluted with the complete culture medium at a 1:10 ratio to give the CCK8 assay solution. To the 96-well plate containing the cells (2 × 10^4^ cells per well) was added the CCK8 assay solution (10 μL per well). The plate was then incubated at 37 °C in the dark for 1 h. The absorbance at 450 nm (OD_450_) was measured to determine the cell viability.

#### 2.5.5 Hematoxylin and eosin (H&E) staining

A 1 cm segment of the intestinal tissue was taken at 20 cm from the gyrus and fixed in 4 % paraformaldehyde for 24 h. After the tissue sample was dehydrated with a sucrose gradient (20 % and 30 %), it was embedded in paraffin and sectioned into 4 μm slices for standard H&E staining. Histopathological injury was quantified using Chiu's scoring system.

#### 2.5.6 Immunofluorescence staining

The cell suspension obtained after the digestion of cells was added to a 24-well plate (1 × 10^5^ cells/mL, 500 μL/well) containing one circular slide in each well. The cells were incubated according to the cell model setup (under OGD/R and/or with IQ) described in Section 2.3. Afterwards, the slides were gently rinsed with PBS three times, immersed in a staining vat containing 4 % paraformaldehyde and incubated at 4 °C for 15 min, blocked with 10 % bovine serum albumin (BSA) for 1 h, incubated with the primary antibodies overnight at 4 °C and then with the secondary antibody at room temperature for 2 h, and finally stained with DAPI in the dark for 10 min and shielded with an anti-fluorescence quencher. Images were then taken by fluorescence microscopy (Nikon, Tokyo, Japan) and analyzed semi-quantitatively using ImageJ. The primary antibodies included IL-1β (26048-1-AP, 1:200), Nrf2 (16396-1-AP, 1:200), HO-1 (10701-1-AP, 1:200), NLRP3 (30109-1-AP, 1:200), and ZO-1 (21773-1-AP, 1:1000), all purchased from Proteintech (Rosemont, IL, USA). The secondary antibodies were Alexa Fluor 488 Goat Anti-Rabbit IgG secondary antibody (A0423, 1:500) and Cy3-labeled Goat Anti-Rabbit IgG secondary antibody (A0521, 1:500), both purchased from Beyotime.

#### 2.5.7 Cytokine assays

When the mice were euthanized, blood was collected into EP tubes containing sodium heparin. The tubes were centrifuged at 4 °C and 1000*g* for 15 min, and the obtained serum was preserved at −80 °C until analysis. All serum samples were assayed using the Mouse Inflammation Panel 1 kit (RayBiotech Biotechnology Co., Ltd., Guangzhou, China) according to the manufacturer's protocol. The cytokine levels were quantified by flow cytometry.

#### 2.5.8 ROS

An ROS detection kit (S0034 M; Beyotime) was used to measure the ROS levels. Cells were seeded in 96-well plates, treated with DCFH-DA (10 μM), and then incubated in a humidified environment (5 % CO_2_, 37 °C) in the dark for 20 min. The absorbance of each well was measured using a Varioskan LUX multimode microplate reader (Thermo Fisher Scientific, Waltham, MA, USA) or by fluorescence microscopy.

#### 2.5.9 Measurement of superoxide dismutase (SOD), malondialdehyde (MDA), and reduced-to-oxidized glutathione ratio (GSH/GSSG)

The levels of SOD, MDA, and the GSH/GSSG ratio in small intestinal tissues and Caco-2 cells were quantified using commercial assay kits (SOD, S0101S; MDA, S0131S; GSH/GSSG, S0053; all from Beyotime) according to the manufacturer's protocols. Specifically, the MDA content was determined by measuring the absorbance at 532 nm with a spectrophotometer. The SOD activity and GSH/GSSG ratio were assessed by measuring the absorbance at 450 nm and 412 nm, respectively, using a Varioskan LUX multimode microplate reader (Thermo Fisher).

### 2.6 Statistical analysis

Statistical analysis was performed using GraphPad Prism 9.0.1 (GraphPad Software Inc., San Diego, CA, USA). Data are presented as the mean ± standard error of the mean (SEM) from three independent experiments. For comparisons between two groups, independent samples *t*-test or paired *t*-test was used, as appropriate. For comparisons involving three or more groups, one-way analysis of variance (ANOVA) was employed. Following a significant ANOVA result, post-hoc pairwise comparisons were conducted using Tukey's multiple comparisons test. The threshold of statistical significance was set at *P* < 0.05.

## 3 Results

### 3.1 IQ attenuated II/R-induced intestinal damage

**Fig. 2:**
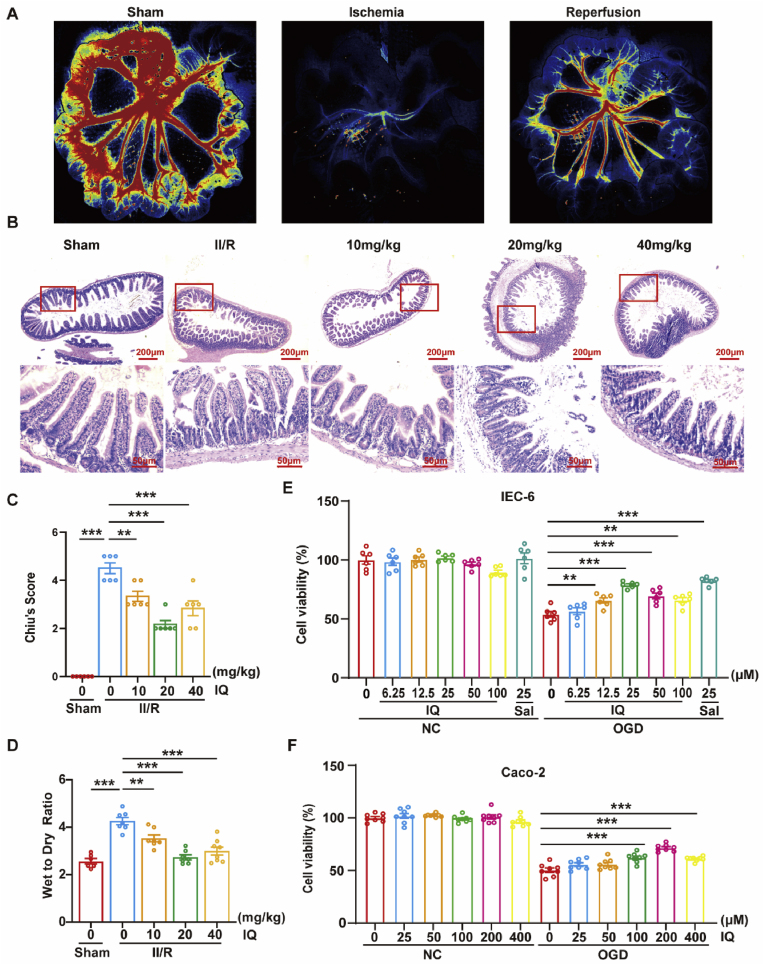
IQ attenuated II/R-induced intestinal damage. (A) Representative laser scatter plots of intestinal tissue in the II/R models. (B) H&E staining of intestinal tissue sections from mouse models. Scale bars: 200 μm (top), 50 μm (bottom). (C) Chiu's scores of intestinal tissue injury (*n* = 6 pooled mice per group; *F*(4, 25) = 64.29). (D) Intestinal tissue weight loss ratio in mouse models (*n* = 6–8 pooled mice per group, *F*(4, 30) = 22.24). (E) Viability of IEC-6 cells treated with IQ under NC or OGD/R conditions. Salidroside (Sal) served as the experimental positive control (*n* = 6 biologically independent samples per group, NC: *F*(6, 35) = 2.287, OGD: *F*(6, 35) = 23.61). (F) Viability of Caco-2 cells treated with IQ under NC or OGD/R conditions (*n* = 8 biologically independent samples per group, NC: *F*(5, 42) = 1.775, OGD: *F*(5, 42) = 21.29). Data were pooled from at least three independent experiments. Values are shown as mean ± SEM. ∗∗*P* < 0.01, ∗∗∗*P* < 0.001.

The mice in Groups M3, M4, and M5 received a daily intraperitoneal injection of IQ at the corresponding dose for four consecutive days before the surgery. The dosage of the injection, set at 10, 20, and 40 mg/kg, was selected based on prior studies [15,17]. Following ischemic insult, the intestinal vasculature developed complete vascular occlusion, with subsequent ligation release inducing only segmental tissue reperfusion, which suggested persistent microcirculatory compromise (Fig. 2A). Histopathological analysis using H&E staining combined with Chiu's scoring system demonstrated that II/R resulted in significant structural disruption of the intestinal architecture (Fig. 2B and C). Compared to the mice receiving sham surgery (Group M1), those with II/R (Group M2) exhibited villous disorganization, serious edema, severe structural breaks, and notable epithelial villi detachment in the morphology of their intestinal sections. However, when the mice were treated with 20 or 40 mg/kg IQ before the surgery (Groups M4 and M5), the intestinal mucosal damage was reduced, and edema and villi breakage were mitigated. The weight loss ratio (γ) of the intestinal tissue reflected the degree of edema and thus the severity of intestinal damage. Fig. 2D shows that II/R significantly increased γ and IQ significantly decreased γ. Thus, IQ could alleviate intestinal edema and reduce II/R injury.

Fig. 2E–F shows the results of the *in vitro* experiments with IEC-6 and Caco-2 cells. According to the CCK8 assay results, IQ was not cytotoxic under normal conditions, as culturing IEC-6 cells with up to 100 μM IQ or Caco-2 cells with up to 400 μM IQ did not change the cell viability significantly. On the other hand, OGD/R significantly decreased the cell viability of both cell lines, but the damage was mitigated by IQ in a dose-dependent manner. For IEC-6 cells, the most significant protective effect was observed in OGD/R-injured cells incubated with 25 μM IQ, whereas for Caco-2 cells, the optimal protection was achieved at 200 μM IQ. Consequently, all subsequent *in vitro* experiments used 25 μM IQ for IEC-6 and 200 μM IQ for Caco-2 cells, respectively, for further evaluations.

**Fig. 3:**
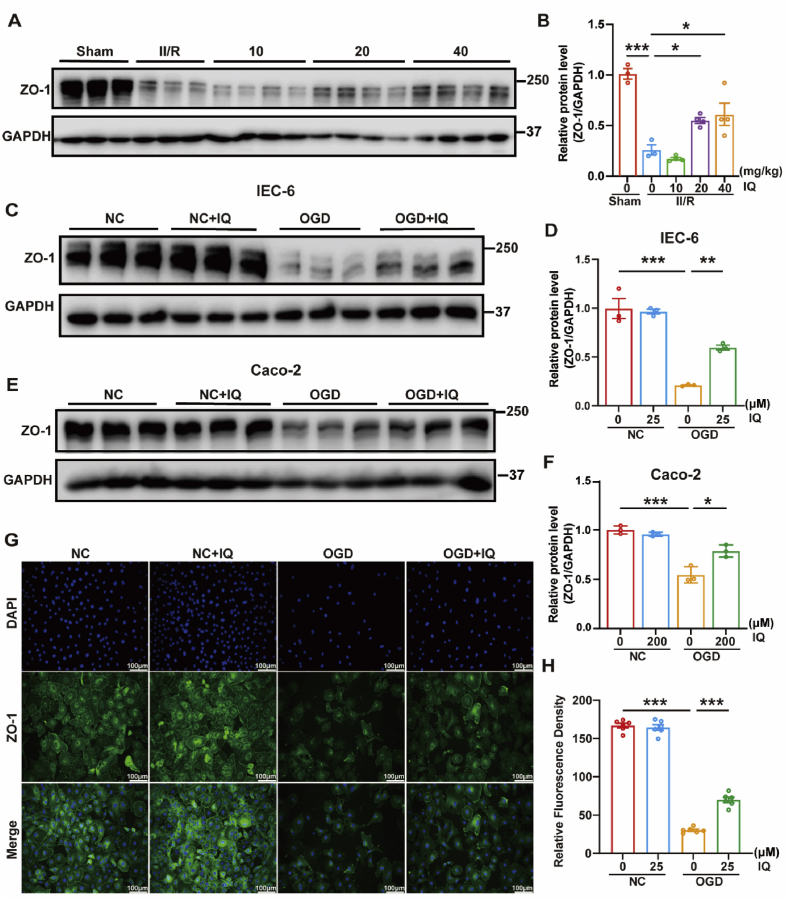
IQ attenuated damage to the intestinal barrier. (A) Western blot images of ZO-1 in intestinal tissue. (B) Quantitative analysis of intestinal ZO-1 protein levels (representative data with *n* = 3–4 pooled mice per group, *F*(4, 13) = 24.79). (C) Western blot images of ZO-1 in IEC-6 cells. (D) Quantitative analysis of ZO-1 protein levels in IEC-6 cells (*n* = 3 biologically independent samples per group, *F*(3, 8) = 40.80). (E) Western blot images of ZO-1 in Caco-2 cells. (F) Quantitative analysis of ZO-1 protein levels in Caco-2 cells (*n* = 3 biologically independent samples per group, *F*(3, 8) = 32.57). (G) Representative immunofluorescence images of ZO-1 (green) and DAPI (blue) staining in IEC-6 cells. Scale bar = 100 μm (H) Quantitative analysis of the ZO-1 immunofluorescence staining of IEC-6 cells (*n* = 6 biologically independent samples per group, *F*(3, 20) = 510.4). Data were pooled from at least three independent experiments. Values are shown as mean ± SEM. ∗*P* < 0.05, ∗∗*P* < 0.01, ∗∗∗*P* < 0.001.

Intestinal edema significantly impacts the functions of the intestinal barrier. Under normal physiological conditions, the intestinal mucosal barrier protects the body from endogenous substances and toxins by blocking the diffusion of intestinal bacteria and confining their toxins within the intestinal lumen [33,34]. Fig. 3 shows that IQ mitigated the damage to the intestinal barrier caused by II/R. ZO-1 is a submembrane protein that, together with other tight junction proteins, forms intercellular junctional complexes [37]. The ZO-1 level was dramatically reduced by the II/R injury but recovered when the mice received IQ, most notably at 20 and 40 mg/kg (Fig. 3A and B). For the IEC-6 and Caco-2 cells, the ZO-1 level fell when the cells were subjected to OGD/R but rebounded when they were incubated with IQ (Fig. 3C–F). When the IEC-6 cells experienced OGD/R, intercellular gaps formed, indicating barrier compromise. In contrast, the IEC-6 cells exhibited better intercellular connections if they were incubated with IQ while under OGD/R (Fig. 3G and H). Thus, it could be inferred that IQ could effectively alleviate the damage to the intestinal barrier induced by the II/R injury and restore its integrity.

### 3.2 IQ improved the structure of the intestinal flora

**Fig. 4:**
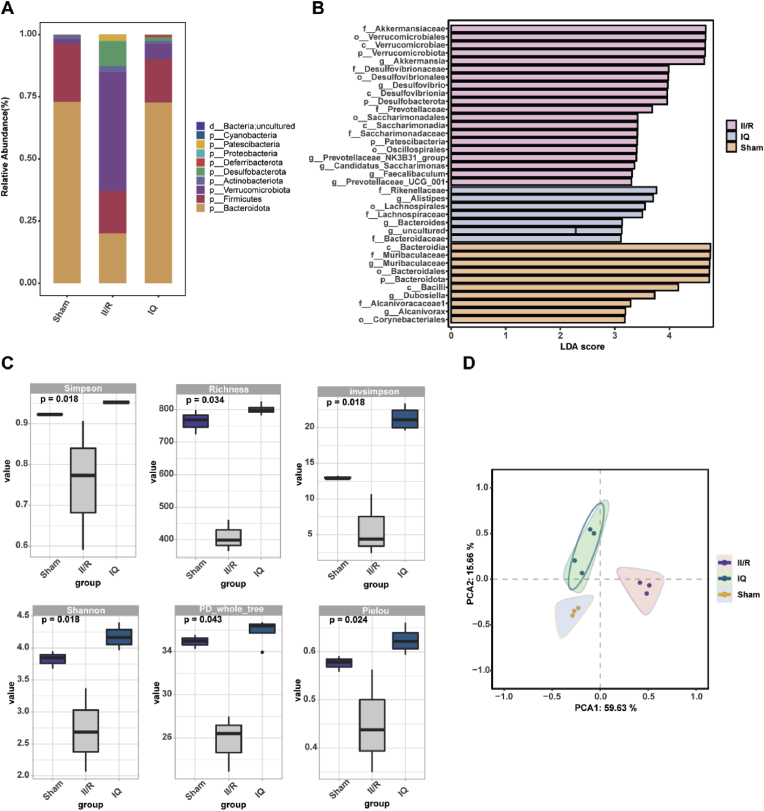
Effects of IQ on the intestinal flora. (A) Relative abundance at the phylum level. (B) LEfSe of OTUs. (C) Alpha diversity indexes. (D) PCoA based on the altGower distance. Consistent results were obtained in three independent experiments; representative data are shown with *n* = 3–4 pooled mice per group. Values are shown as mean ± SEM.

The intestinal flora (also known as the intestinal microbiota) is the largest symbiotic ecosystem in the human body. Some pathogenic bacteria in the gut increase the risk of chronic inflammation and gut-related diseases, as they suppress the activity of immune cells and damage the intestinal mucosal barrier. The 16S rRNA sequencing showed that II/R dramatically affected the gut microbiota. While the II/R injury increased the abundance of Verrucomicrobiota and decreased that of Bacteroidota and Firmicutes, the administration of IQ mitigated these effects, as it reduced the abundance of Verrucomicrobiota and increased the abundance of Bacteroidota. Although IQ did not increase the relative abundance of Firmicutes, it helped to decrease the Firmicutes/Bacteroidota ratio despite the II/R injury, thus improving the intestinal microenvironment. The abundance of the operational taxonomic units (OTUs) showed that the intestinal flora became disorganized upon II/R, but IQ alleviated the disorganization (Fig. 4A). The bacterial taxa that significantly differed among treatment groups were identified by LEfSe (Fig. 4B), and the highly abundant taxa for each group were as follows: Group M1 (sham surgery), p__Bacteroidota, o__Bacteroidales, g__Muribaculaceae, f__Muribaculaceae, and c__Bacteroidia (LDA >4); Group M2 (II/R injury without any IQ treatment), Akkermansiaceae, Verrucomicrobiales, Verrucomicrobiae, and Verrucomicrobiota (LDA >4); Group M4 (II/R injury with IQ treatment at 20 mg/kg), o__Lachnospirales, g__Alistipes, and f__Rikenellaceae (LDA >3).

**Fig. 5:**
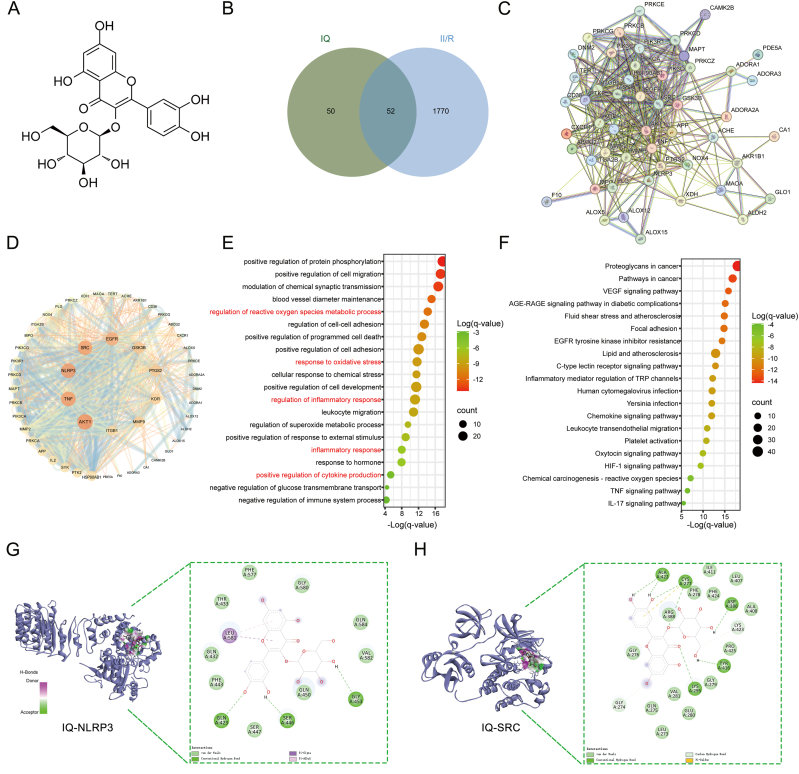
Pharmacological analysis of the IQ-II/R network. (A) The chemical structure of IQ. (B) Venny figure. (C, D) Protein–protein interaction network. (E) GO enrichment analysis. (F) KEGG enrichment analysis. (G) Molecular docking of IQ-NLRP3. (H) Molecular docking of IQ-SRC.

There were significant variations among treatment groups in the alpha diversity indices of the intestinal microbiota. Fig. 4C shows that the II/R injury significantly reduced the Shannon index, Simpson index, Invsimpson index, Pielou index, and PD_whole_tree index of the intestinal flora, but the IQ treatment significantly increased these indexes. That is, the II/R injury had a substantial impact on the number and diversity of the intestinal microbiota in mice. On the other hand, IQ exerted a positive regulatory effect and boosted the abundance and diversity of the intestinal microbiota to some extent. Fig. 4D shows that the differences in the intestinal flora between treatment groups were clearly identified by principal coordinate analysis (PCoA). Hence, IQ could substantially influence the diversity and composition of the intestinal flora, thus improving the intestinal milieu. This, in turn, can reduce the disruption to the intestinal flora caused by II/R injury and protect the intestinal mucosal barrier.

### 3.3 Network pharmacological target prediction

A total of 102 potential therapeutic targets for IQ were identified through database screening. Using Gene Cards, OMIM, and DrugBank databases, we obtained 1822 II/R targets. The 52 overlapping targets were identified as putative mediators of IQ's therapeutic effects in II/R injury (Fig. 5B). A protein–protein interaction network of intersecting targets was constructed using String, comprising 52 nodes and 412 interactions. Core target proteins included AKT1, TNF, NLRP3, SRC, and EGFR (Fig. 5C and D). GO enrichment analysis of the 52 potential target genes highlighted biological processes such as positive regulation of protein phosphorylation, positive regulation of cell migration, modulation of chemical synaptic transmission, maintenance of blood vessel diameter, regulation of reactive oxygen species metabolic process, regulation of cell-cell adhesion, positive regulation of programmed cell death, positive regulation of cell adhesion, and regulation of inflammatory response (Fig. 5E). KEGG pathway enrichment analysis indicated that IQ's mechanism for preventing and treating II/R involved 145 pathways, including proteoglycans in cancer, pathways in cancer, VEGF signaling pathway, AGE-RAGE signaling pathway in diabetic complications, lipid and atherosclerosis, inflammatory mediator regulation of TRP channels, chemokine signaling pathway, leukocyte transendothelial migration, and platelet activation (Fig. 5F). Based on literature research, we hypothesized that IQ may mitigate II/R injury by influencing redox-related signaling pathways and regulating NLRP3-mediated inflammatory responses.

Molecular docking using Autodock Vina determined the binding affinities of IQ with five hub proteins (AKT1, TNF, NLRP3, SRC, EGFR), with values of −4.98, −3.77, −3.46, −6.57, and −7.37 kcal/mol, respectively. The docking results indicate that IQ interacts well with multiple amino acid residues in the SRC binding pocket (Fig. 5G and H). In ischemic diseases, SRC activation has been found to correlate with changes in ROS levels, and the Nrf2/HO-1 signaling pathway is considered a crucial pathway related to oxidative stress. Therefore, for the validation of both *in vitro* and *in vivo* experiments, we selected the classical Nrf2/HO-1 signaling pathway and the inflammatory response triggered by the activation of the NLRP3 inflammasome.

### 3.4 IQ inhibited NLRP3-mediated inflammatory response after ischemia

**Fig. 6:**
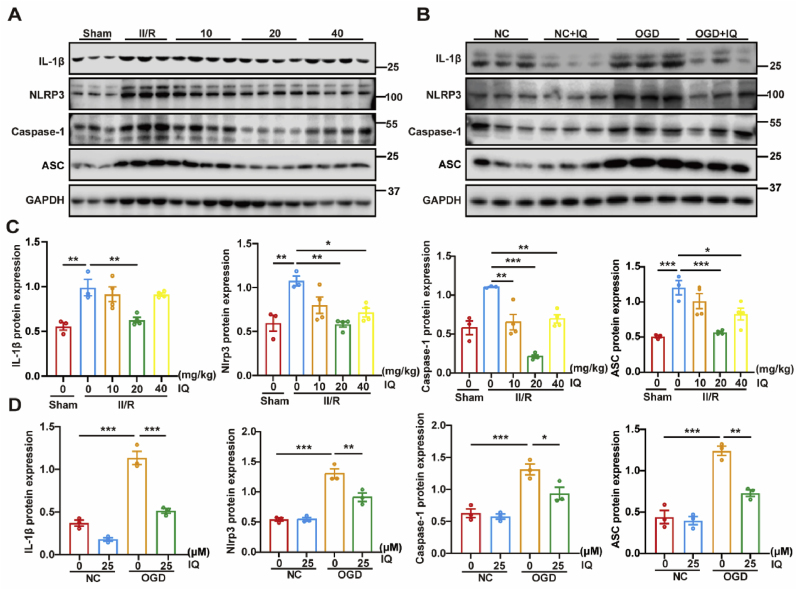
Effects of IQ on the NLRP3 inflammasome. (A) Western blot images of NLRP3, Caspase-1, ASC, and IL-1β in intestinal tissue. (B) Western blot images of NLRP3, Caspase-1, ASC, and IL-1β in IEC-6 cells. (C) Quantitative analysis of NLRP3, Caspase-1, ASC, and IL-1β protein levels in intestinal tissue (representative data with *n* = 3–4 pooled mice per group; IL-1β: *F*(4, 13) = 10.46; NLRP3: *F*(4, 13) = 7.793; Caspase-1: *F*(4, 13) = 21.83; ASC: *F*(4, 13) = 12.64). (D) Quantitative analysis of NLRP3, Caspase-1, ASC, and IL-1β protein levels in IEC-6 cells (*n* = 3 biologically independent samples per group; IL-1β: *F*(3, 8) = 79.67; NLRP3: *F*(3, 8) = 40.04; Caspase-1: *F*(3, 8) = 19.92; ASC: *F*(3, 8) = 43.96). Data were pooled from at least three independent experiments. Values are shown as mean ± SEM. ∗*P <* 0.05, ∗∗*P <* 0.01, ∗∗∗*P* < 0.001.

**Fig. 7:**
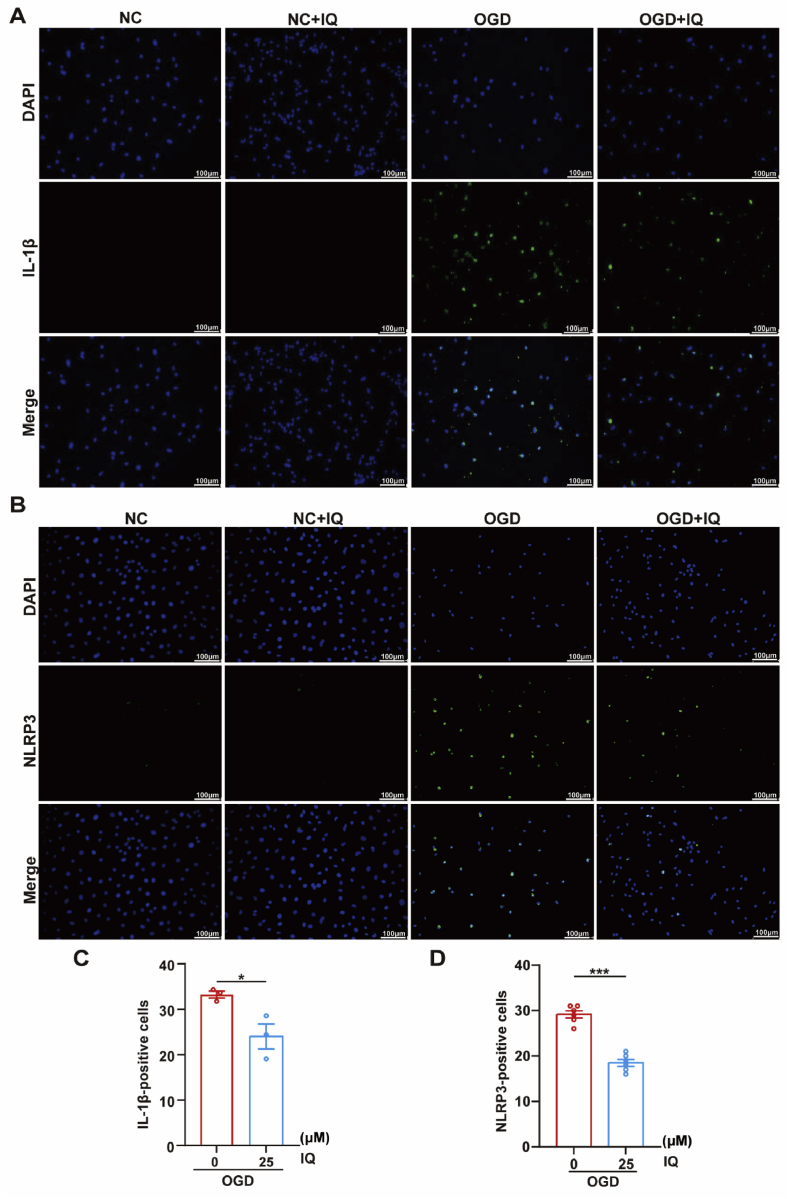
Effects of IQ on the NLRP3 inflammasome in OGD/R-injured IEC-6 cells. (A) Representative immunofluorescence images of IL-1β (green) and DAPI (blue) staining. Scale bar = 100 μm (B) Representative immunofluorescence images of NLRP3 (green) and DAPI (blue) staining. Scale bar = 100 μm (C) Quantitative analysis of IL-1β immunofluorescence intensity (*n* = 3 biologically independent samples per group). (D) Quantitative analysis of NLRP3 immunofluorescence intensity (*n* = 6 biologically independent samples per group). Data were pooled from at least three independent experiments. Values are shown as mean ± SEM. ∗*P* < 0.05, ∗∗∗*P* < 0.001.

The inflammatory response is an evident pathological process in II/R injury [37]. In the mouse models, the intestinal tissue with II/R injury exhibited increased expression of NLRP3-related proteins including NLRP3, Caspase-1, and ASC, along with a significant upregulation of the cytokine IL-1β. The IQ treatment substantially inhibited the upregulation of these proteins, and the most significant impact was noted in the mice treated with 20 mg/kg IQ (Fig. 6A and C). In the cell models, the cells subjected to OGD/R showed increased positive staining in the immunofluorescence staining for IL-1β and NLRP3, but this increase was tapered by the administration of IQ (Fig. 7). The Western blot experiments yielded comparable results (Fig. 6B and D). It could be inferred that the II/R injury activated the NLRP3 inflammasome, but IQ significantly attenuated the activation and the associated intestinal inflammation.

### 3.5 IQ inhibited the expression of intestinal cytokines

Cytokines are key proteins in cell signaling and exert significant influences on the modulation of immune and inflammatory responses. The chemokine IL-6 is mainly derived from vascular endothelial cells, monocyte macrophages, fibroblasts, etc., and the chronic overproduction of IL-6 is associated with autoimmune diseases, chronic inflammatory conditions, and cancers. The cytokine IL-4, which is derived from type 2 helper T cells, is an immune modulator that enhances the activity of immune cells (B cells, T cells) in the organism. Keratinocyte-derived cytokines (KC) can activate a variety of immune cells, and they are known to act as sentinels, mediate intercellular communication, and produce antimicrobial peptides. The dysregulation of these cytokines can lead to various pathological conditions.

**Fig. 8:**
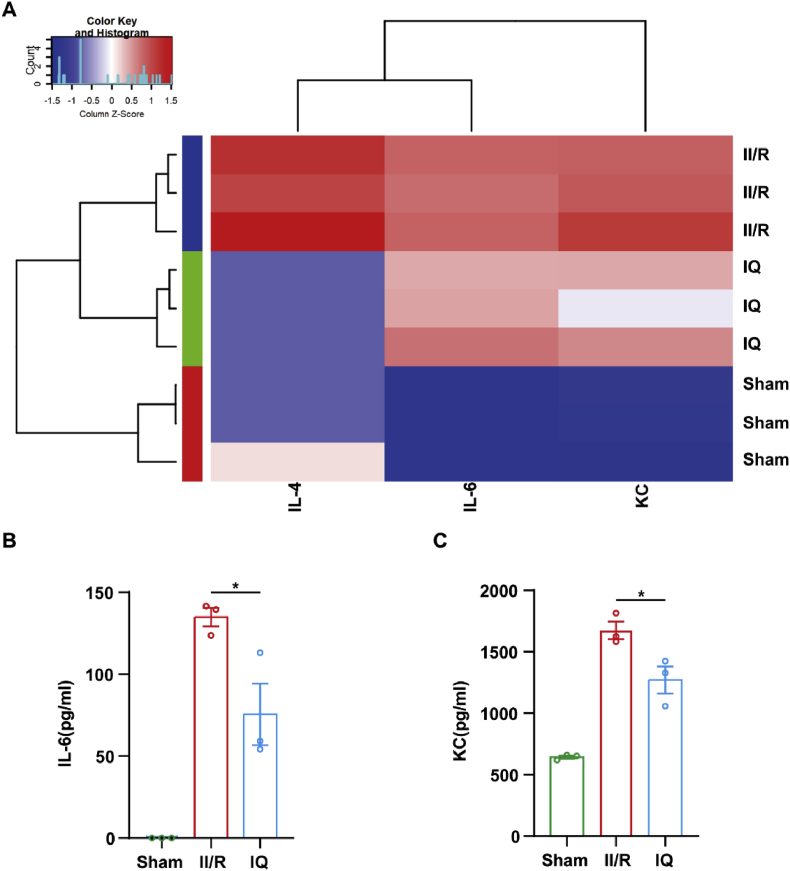
Effects of IQ on the peripheral blood inflammatory factors in mice. (A) Heatmap of serum cytokine levels in mice. (B) Quantitative analysis of serum IL-6 levels (*n* = 3 pooled mice per group). (C) Quantitative analysis of serum KC levels (*n* = 3 pooled mice per group). Data are representative of or pooled from three independent experiments. Values are expressed as mean ± SEM. ∗*P* < 0.05.

The cytokine/chemokine levels in the plasma were evaluated by flow cytometry to assess the effects of IQ on the peripheral cytokine levels. Fig. 8 shows that in the mouse models, the serum concentrations of IL-6 and KC were significantly elevated after II/R injury but were reduced when IQ was administered (20 mg/kg). Thus, the IQ treatment greatly decreased the levels of cytokines in the peripheral region and attenuated the inflammatory response to the II/R injury.

### 3.6 IQ reduced oxidative stress

Alterations in oxidative stress are common in II/R-related pathological processes [38]. MDA is a naturally occurring substance that is produced during lipid oxidation in mammalian systems, and the production increases with rising oxidative stress. SOD, a potent antioxidant enzyme, scavenges free radicals, thereby mitigating oxidative stress. Glutathione (GSH) and its oxidized form, glutathione disulfide (GSSG), are key bioactive substances in animal cells and play a crucial role in maintaining cellular redox homeostasis. Consequently, MDA levels, SOD activity, and the GSH/GSSG ratio serve as reliable biomarkers of oxidative stress.

**Fig. 9:**
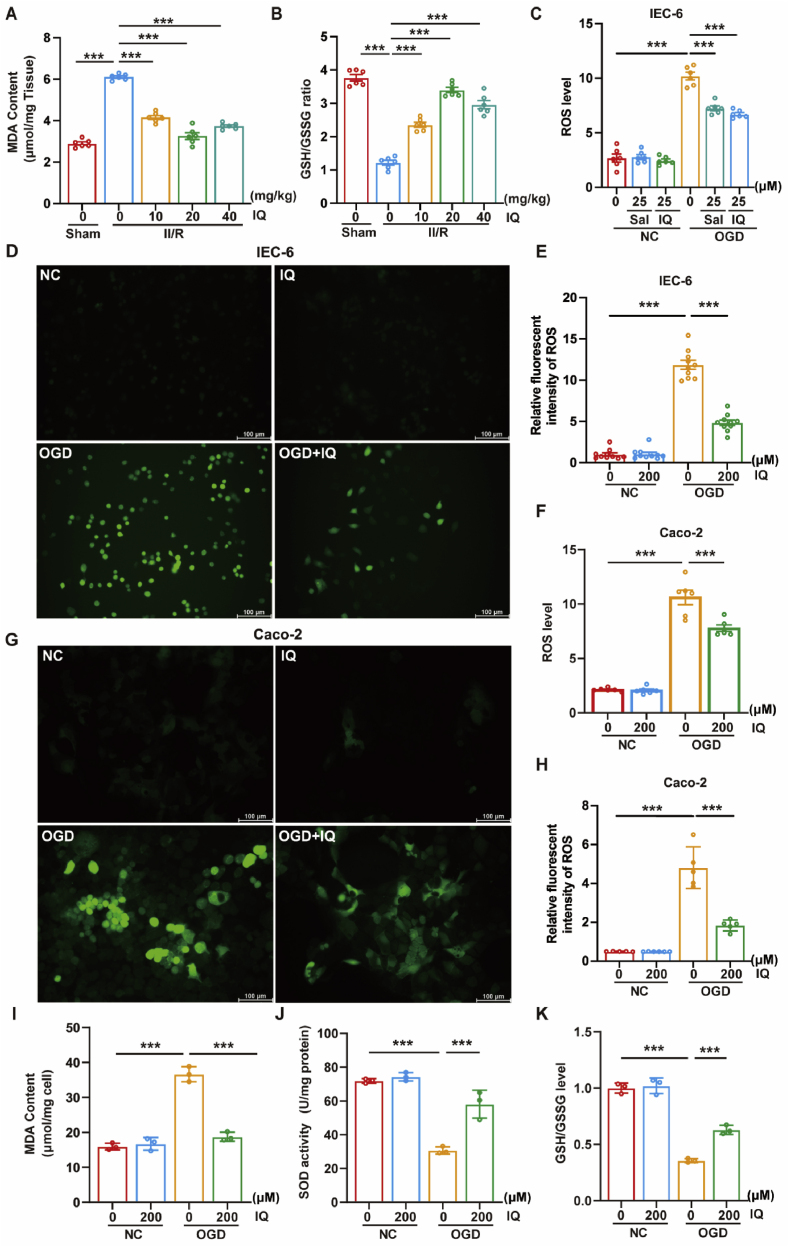
IQ inhibited oxidative stress. (A) The MDA level (*F*(4, 25) = 163.6) and (B) the GSH/GSSG ratio (*F*(4, 25) = 120.2) of the intestinal tissue (representative data with *n* = 6 pooled mice per group). (C) The intracellular ROS in IEC-6 cells determined by enzymatic assay. Salidroside (Sal) served as the experimental positive control (*n* = 6 biologically independent samples per group, *F*(5, 30) = 154.5). (D) Immunofluorescence detection of the intracellular ROS in IEC-6 cells. Scale bar = 100 μm (E) Quantitative analysis of the ROS-positive IEC-6 cells (*n* = 10 biologically independent samples per group, *F*(3, 36) = 211.6). (F) The intracellular ROS in Caco-2 cells determined by enzymatic assay (*n* = 6 biologically independent samples per group, *F*(3, 20) = 130.6). (G) Immunofluorescence detection of the intracellular ROS in Caco-2 cells. Scale bar = 100 μm (H) Quantitative analysis of ROS-positive Caco-2 cells (*n* = 5 biologically independent samples per group, *F*(3, 17) = 73.98). (I) The MDA level (*F*(3, 8) = 109.5), (J) the SOD activity (*F*(3, 8) = 59.98), and (K) the GSH/GSSG ratio (*F*(3, 8) = 141.6) in Caco-2 cells (*n* = 3 biologically independent samples per group) Data were pooled from at least three independent experiments. Values are expressed as mean ± SEM. ∗∗∗*P <* 0.001.

In the mouse models, the II/R injury increased the MDA level while reducing the GSH/GSSG ratio, which corresponded to elevated oxidative stress. When the mice received IQ, the MDA level dropped and the GSH/GSSG ratio recovered (Fig. 9A and B), which indicated lower oxidative stress. In the cell models, OGD/R significantly increased the ROS level, but IQ attenuated the rise of ROS (Fig. 9C and F), and the immunofluorescence results matched the enzyme activity assay results (Fig. 9D,E,G,H). Furthermore, IQ significantly suppressed the MDA level and increased the SOD activity and the GSH/GSSG ratio in the Caco-2 cell model (Fig. 9I–K). Thus, IQ could effectively inhibit the oxidative stress caused by the II/R injury.

### 3.7 IQ activated the Nrf2/HO-1 signaling pathway

**Fig. 10:**
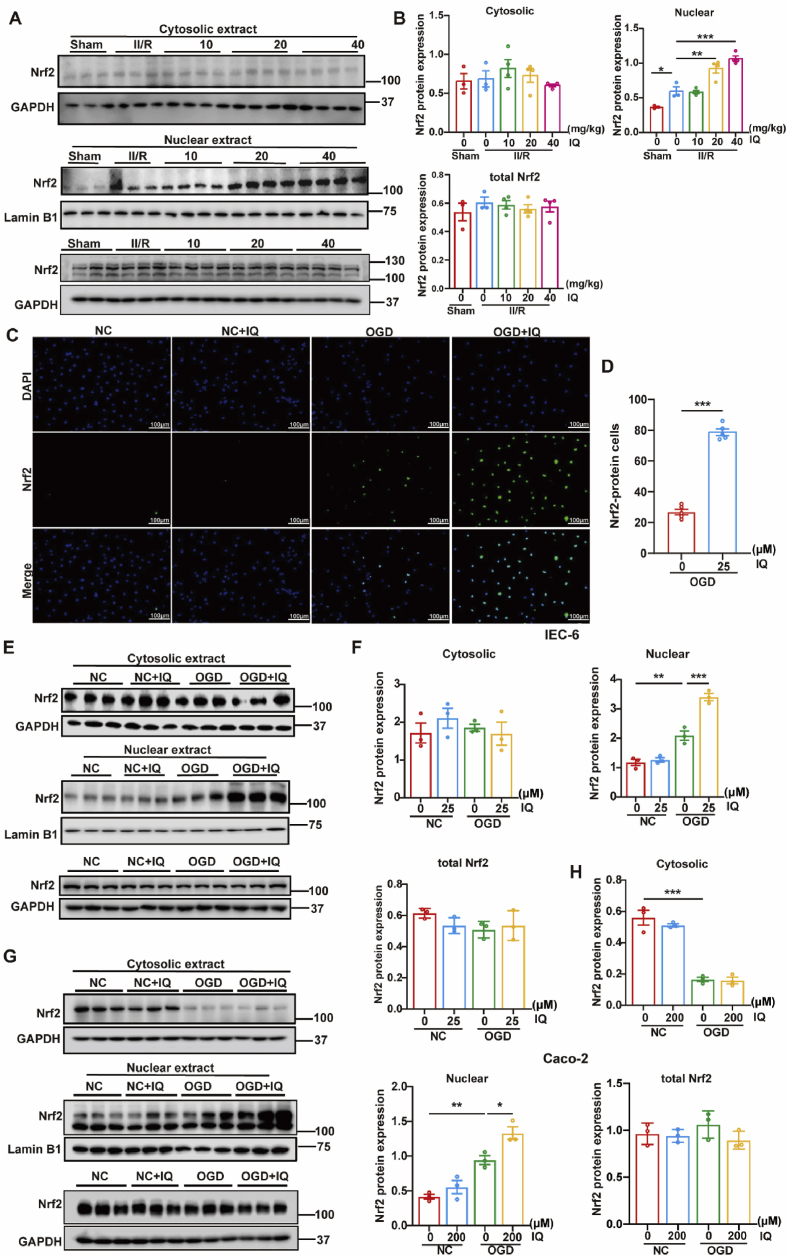
Effects of IQ on Nrf2. (A) Western blot images of Nrf2 in intestinal tissue. (B) Quantitative analysis of Nrf2 levels in intestinal tissue (nuclear: *F*(4,13) = 36.30; total: *F*(4,13) = 0.3981; *n* = 3–4 pooled mice per group; data are representative of three independent experiments). (C) Representative immunofluorescence images of Nrf2 (green) and nuclei (DAPI, blue) in IEC-6 cells. Scale bar = 100 μm. (D) Quantitative analysis of Nrf2 levels in IEC-6 cells based on immunofluorescence intensity (*n* = 5 biologically independent samples per group). (E) Western blot images of Nrf2 in IEC-6 cells. (F) Quantitative analysis of the Western blot results of Nrf2 levels in IEC-6 cells (cytosolic: *F*(3,8) = 0.5874; nuclear: *F*(3,8) = 72.32; total: *F*(3,8) = 1.635; *n* = 3 biologically independent samples per group). (G) Western blot images of Nrf2 in Caco-2 cells. (H) Quantitative analysis of Nrf2 protein levels in Caco-2 cells (cytosolic: *F*(3,8) = 62.46; nuclear: *F*(3,8) = 30.22; total: *F*(3,8) = 1.248; *n* = 3 biologically independent samples per group). Data were pooled from at least three independent experiments. Values are expressed as mean ± SEM. ∗*P* < 0.05, ∗∗*P* < 0.01, *∗∗∗P* < 0.001.

**Fig. 11:**
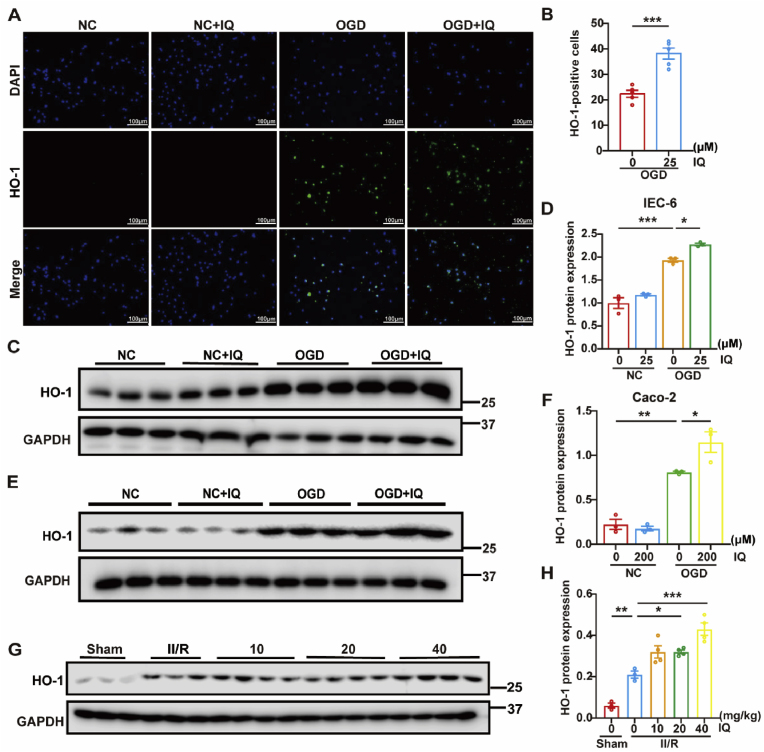
Effects of IQ on HO-1. (A) Detection of HO-1 levels by HO-1 immunofluorescence staining (green) and DAPI staining (blue), scale bar = 100 μm (B) Quantitative analysis of HO-1 immunofluorescence staining in IEC-6 cells (*n* = 4 biologically independent samples per group). (C) Western blot images of HO-1 in IEC-6 cells. (D) Quantitative analysis of HO-1 protein levels in IEC-6 cells (*n* = 3 biologically independent samples per group, *F*(3,8) = 88.88). (E) Western blot images of HO-1 in Caco-2 cells. (F) Quantitative analysis of HO-1 protein levels in Caco-2 cells (*n* = 3 biologically independent samples per group, *F*(3,8) = 51.58). (G) Western blot images of HO-1 in intestinal tissue. (H) Quantitative analysis of HO-1 protein levels in intestinal tissue (*n* = 3–4 pooled mice per group, *F*(3,8) = 33.86). Data were pooled from at least three independent experiments. Values are expressed as mean ± SEM. ∗*P <* 0.05, ∗∗*P <* 0.01, ∗∗∗*P <* 0.001.

The Nrf2/HO-1 axis is intimately involved in the ischemia/reperfusion (I/R) injury of various organs [27]. The nuclear and plasma proteins in the intestinal tissue of the mouse models were isolated and analyzed to examine the impacts of IQ on Nrf2. The intranuclear expression of Nrf2 was significantly increased after II/R, and IQ further increased the nuclear Nrf2 levels in a dose-dependent manner (Fig. 10A and B). Both immunofluorescence staining and Western blot revealed that in the cell models, Nrf2 was significantly activated and rapidly translocated to the nucleus when the cells were subjected to OGD/R, and IQ further promoted this nuclear translocation (Fig. 10C–H). The level of the antioxidant protein HO-1 was also significantly increased after II/R (*in vivo*) or OGD/R (*in vitro*), and it was increased further by the IQ treatment (Fig. 11). It could be speculated, based on the observed nuclear translocation of Nrf2 and changing HO-1 level, that IQ mitigated the II/R injury via the Nrf2/HO-1 axis.

### 3.8 IQ could no longer protect the intestinal tract from OGD/R injury in the presence of ML385

**Fig. 12:**
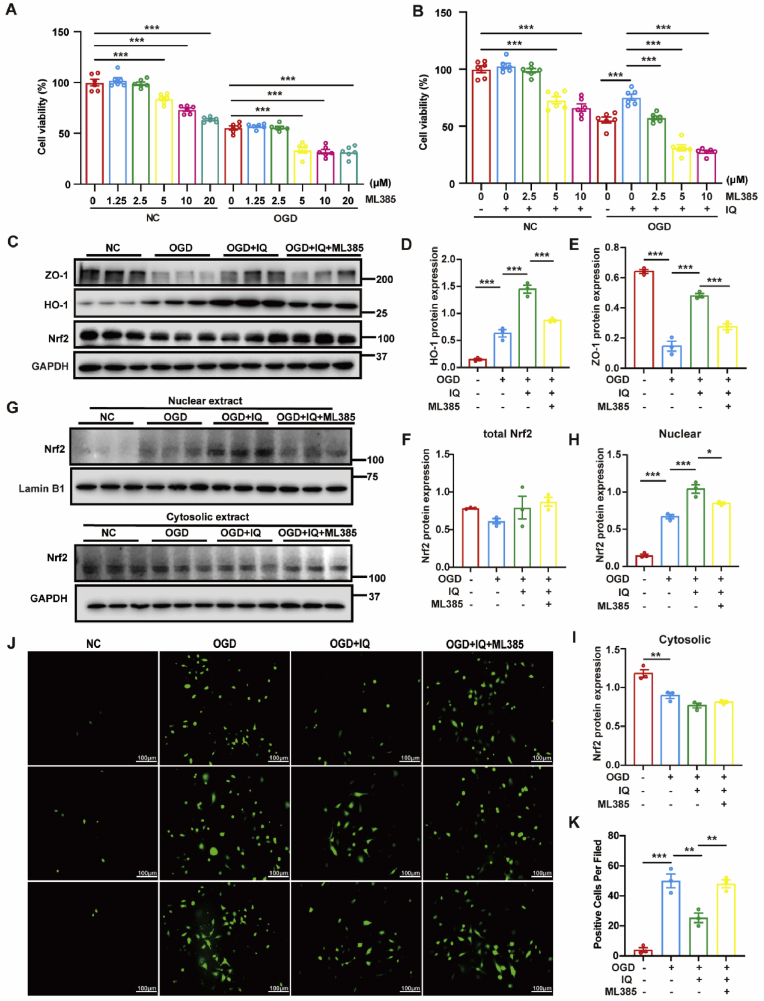
IQ could no longer alleviate the OGD/R injury in the presence of ML385. (A) Viability of IEC-6 cells treated with ML385 without IQ pretreatment (*n* = 6 biologically independent samples per group; NC: *F*(5,30) = 55.84; OGD: *F*(5,30) = 42.84). (B) Viability of IEC-6 cells treated with ML385 with IQ pretreatment (*n* = 6 biologically independent samples per group; NC: *F*(4,25) = 38.50; OGD: *F*(4,25) = 78.28). (C) Western blot images of HO-1, ZO-1, and total Nrf2 in IEC-6 cells. (D–F) Quantitative analysis of (D) HO-1 (*F*(3,8) = 108.9), (E) ZO-1 (*F*(3,8) = 110.6), and (F) total Nrf2 (*F*(3, 8) = 1.709) levels (*n* = 3 biologically independent samples per group). (G) Western blot images of Nrf2 in the nucleus and cytoplasm of IEC-6 cells. (H, I) Quantitative analysis of Nrf2 levels in the (H) nucleus (*F*(3,8) = 131.0) and (I) cytoplasm (*F*(3,8) = 30.95) of IEC-6 cells (*n* = 3 biologically independent samples per group). (J) Representative immunofluorescence images of intracellular ROS in IEC-6 cells (scale bar = 100 μm) (K) Quantitative analysis of intracellular ROS levels (*n* = 3 biologically independent samples per group, *F*(3,8) = 46.05). Data were obtained from at least three independent experiments. Values are expressed as mean ± SEM. ∗*P* < 0.05, ∗∗*P* < 0.01, ∗∗∗*P* < 0.001.

Additional experiments were conducted using cell models to clarify if IQ indeed stimulated the Nrf2/HO-1 axis to alleviate the OGD/R injury. First, the Nrf2 inhibitor ML385 was administered to IEC-6 cells at varying concentrations (1.25–20 μM) to determine its impact on the cell viability under normal conditions and after OGD/R injury. Fig. 12A shows that under normal conditions, the cell viability was significantly reduced when the concentration of ML385 was 5 μM or higher. Fig. 12B shows that when the cells were exposed to both 25 μM IQ and various concentrations of ML385, the cell viability decreased significantly when the ML385 concentration exceeded 2.5 μM. Thus, the toxicity threshold of ML385 was considered 2.5 μM. Fig. 12C–I shows that ML385 counteracted IQ in regulating the nuclear translocation of Nrf2, the HO-1 level, and the ZO-1 level. In addition, in the presence of ML385, IQ could no longer strongly inhibit the accumulation of intracellular ROS (Fig. 12J and K). Since inhibiting Nrf2 largely cancelled the protective effect of IQ, it could be confirmed that IQ reduced the OGD/R injury through the Nrf2/HO-1 signaling pathway.

### 3.9 Nrf2 knockdown abolished IQ's protective effects against OGD/R injury in Caco-2 cells

**Fig. 13:**
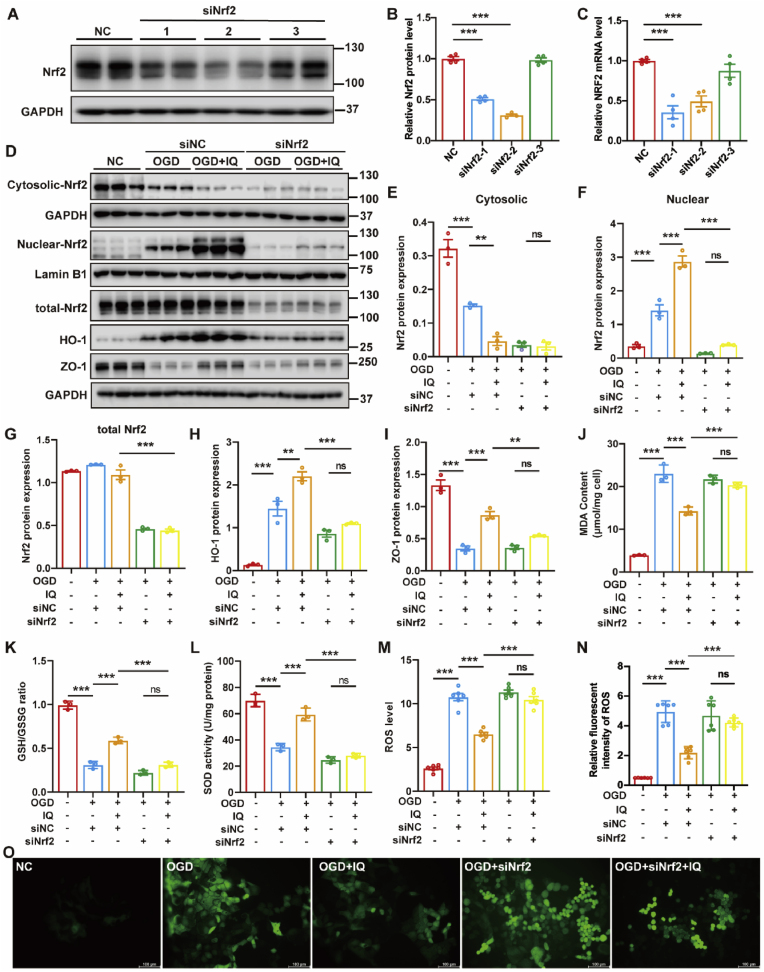
Nrf2 knockdown abolished IQ's protective effects against OGD/R injury in Caco-2 cells. (A–C) Knockdown of Nrf2 protein (A, B; *F*(3,12) = 248.8) and mRNA expression (C; *F*(3,12) = 20.63) using siNrf2 in Caco-2 cells was confirmed by Western blotting and RT-PCR (*n* = 4 biologically independent samples per group). (D–I) Western blot analysis of Nrf2 subcellular localization (nuclear: *F*(4,10) = 116.1; cytoplasmic: *F*(4,10) = 73.18), total Nrf2 (*F*(4,10) = 207.2), HO-1 (*F*(4,10) = 61.23), and ZO-1 (*F*(4,10) = 69.63) levels in siNrf2-transfected Caco-2 cells (*n* = 3 biologically independent samples per group). (J–L) Levels of MDA (J; *F* (4, 10) = 146.5), GSH/GSSG ratio (K; *F* (4, 10) = 221.7), and SOD activity (L; *F* (4, 10) = 96.21) in Caco-2 cells (*n* = 3 biologically independent samples per group). (M) Intracellular ROS levels determined by enzymatic assay (*F*(4,25) = 158.6; *n* = 6 biologically independent samples per group). (N) Quantification of intracellular ROS immunofluorescence (*F*(4,25) = 62.16; *n* = 6 biologically independent samples per group). (O) Representative immunofluorescence images of intracellular ROS in Caco-2 cells. Scale bar = 100 μm. Data were obtained from at least three independent experiments. Values are expressed as mean ± SEM. ns, not significant, ∗∗*P* < 0.01, ∗∗∗*P* < 0.001.

To further verify Nrf2-dependency, we designed an Nrf2-targeting siRNA, which effectively reduced the mRNA and protein levels of Nrf2 in Caco-2 cells (Fig. 13A–C). Nrf2 knockdown significantly decreased Nrf2 nuclear translocation and downregulated ZO-1 and HO-1 protein expression, thereby abolishing IQ's protection against OGD/R injury (Fig. 13D–I). Moreover, Nrf2 silencing exacerbated II/R-induced oxidative stress. Following siNrf2 transfection, IQ lost its protective capacity: MDA levels in IQ-treated cells showed no significant reduction compared to OGD/R-damaged cells (Fig. 13J), and the SOD activity and the GSH/GSSG ratio confirmed that Nrf2 ablation attenuated IQ's antioxidant effects (Fig. 13K and L). Notably, IQ failed to suppress ROS overproduction in Nrf2-ablated cells (Fig. 13M − O), which directly linked ROS dysregulation to impaired Nrf2 function. Collectively, these findings demonstrated that the IQ-initiated protection depended on activating the Nrf2 pathway.

## 4 Discussion

II/R injury represents a critical emergency caused by vascular disorders (e.g., trauma, shock, bowel obstruction, mesenteric thrombosis) or surgical interventions (e.g., aortic repair, cardiopulmonary procedures, organ transplantation) [1]. With oxidative stress and inflammation recognized as central drivers of I/R pathogenesis [39,40], and these pathways identified as key targets of IQ through our network pharmacology, IQ demonstrates significant therapeutic potential in mitigating II/R-induced damage. This study, therefore, investigates the effects of IQ on epithelial tight junction integrity, the attenuation of Nrf2/HO-1-mediated oxidative stress, NLRP3 inflammasome activation, and the restoration of gut microbiota homeostasis.

Many IQ-containing plants have been reported to possess important physiological functions. For example, *Eucommia ulmoides* can effectively resist oxidative stress [41]. *Apocynum venetum* can prevent liver damage and platelet clumping [42,43]. *Hypericum* spp. is known for its anti-inflammatory properties [43]. *Cymbopogon flexuosus* can treat high blood cholesterol and improve circulation [44]. *Spondias mombin* L. has anti-anxiety and anti-depression effects [45]. IQ is known as an excellent antioxidant and free radical scavenger. It can reduce the activity of xanthine oxidase and scavenge the superoxide free radicals derived from the xanthine/xanthine oxidase system, inhibit the activity of myeloperoxidase, reduce the ROS level and alleviate the oxidative stress damage in H_2_O_2_-induced rat retinal ganglion cells (RGC-5), prevent carrageenan-induced inflammatory response, and decrease the production of nitric oxide (NO) and iNOS triggered by LPS to reduce neuroinflammation [12]. In the present study, mice developed II/R injury after they experienced the transient occlusion of the SMA, and their intestinal mucosal injury was obvious from both the imaging of the intestinal morphology and the biochemical assay of ZO-1. Gratifyingly, IQ was found to attenuate the II/R-induced damage to the intestinal barrier.

The gut microbiota is also referred to as the “forgotten organ of the human body” [46]. Under normal circumstances, the intestinal tract is populated by diverse and abundant microorganisms, mostly bacteria but also viruses, fungi, and archaea [46,47], that live in symbiosis with the host and contribute to the host's digestion and absorption, nutrient metabolism, immune regulation, and other physiological processes [[46], [47], [48], [49], [50], [51]]. Normally, most of its bacterial taxa (>90 %) are Firmicutes and Bacteroidetes. However, external factors such as dietary habits, bacterial infections, or drug intake can reduce the diversity of the gut microbiota, and certain foods also change the balance of the gut microbiome as their metabolites can affect the growth and reproduction of some microbial species [52]. Meanwhile, endogenous factors such as intestinal congestion, ischemia, hypoxia, acid-base imbalance, and nutritional deficiencies can also change the microecological structure of the gut [53]. The gut microbiota has also been called the “second human genome” due to its vast gene pool, which is roughly 100 times larger than that of the human genome [49]. It has been well correlated with diabetes, hypertension, cancers, cardiovascular disease, etc. The 16S rRNA gene sequencing results in this work demonstrated that the II/R injury significantly altered the gut microbial composition, increasing the abundance of Verrucomicrobia and decreasing the Firmicutes/Bacteroidetes ratio, but IQ could weaken the change brought by II/R. The differences in the intestinal flora could be well distinguished by PCoA for the mice receiving the sham surgery, experiencing the II/R injury without intervention, and undergoing the II/R surgery after having received IQ. In other words, IQ regulated the structure and composition of the intestinal flora in mice, inhibited the proliferation of detrimental bacteria, reduced the translocation of bacteria and their metabolites caused by the II/R injury, to ultimately restore the integrity of the intestinal lining and maintain its function.

In the post-ischemic intestine, the accumulation of ROS exacerbates the peroxidation of lipids in the cell membrane and accentuates the oxidative damage to DNA and proteins, thus intensifying II/R injury [52]. As a product of lipid peroxidation, MDA reflects how seriously cells have been attacked by free radicals, whereas GSH represents the ability of cells to scavenge ROS [32]. The experimental results demonstrated that IQ had antioxidant properties and inhibited II/R-induced ROS accumulation. In the *in vivo* and *in vitro* experiments, the oxidative stress increased after the II/R surgery or OGD/R, which led to elevated MDA production and decreased SOD and GSH activity. However, IQ inhibited the production of ROS and MDA, while increasing SOD and GSH activity.

The NLRP3 inflammasome has been studied extensively [37]. Upon detecting cellular stress signals, NLRP3 assembles the adaptor protein ASC and pro-Caspase-1 to form an inflammasome complex, which results in the activation of Caspase-1. Caspase-1 catalyzes the conversion of pro-inflammatory cytokines, such as IL-1β and IL-18, into their biologically active forms, which are then released from the cell to initiate inflammatory responses and recruit immune cells to the location of infection or damage [54,55]. During II/R injury, damaged cells release danger signals that can activate NLRP3 inflammasomes to trigger the inflammatory cascade. In the present study, IQ alleviated the intestinal inflammatory response that occurred after the II/R injury. Compared to the mice getting sham surgery, those receiving the II/R surgery exhibited markedly higher levels of KC and IL-6, which corresponded to the activation of the NLRP3 inflammasomes, but the levels of those cytokines returned to normal when IQ was administered. In the cell models, the OGD/R treatment elevated the levels of NLRP3, Caspase-1, and IL-1β. However, these effects were effectively counteracted by IQ.

Through network pharmacology, we predicted the therapeutic effects of IQ on II/R. We hypothesized that IQ may serve as a protective factor against II/R injury by modulating redox pathways and inhibiting NLRP3-driven inflammation. The activation of the Nrf2/HO-1 signaling pathway can facilitate the production of downstream antioxidant factors. Nrf2, encoded by the *NFE2L2* gene, is a stress-responsive cap'n'collar (CNC) subfamily of basic-leucine-zipper (bZIP) transcription factor that maintains cellular homeostasis by regulating antioxidant defenses [56]. Normally, Nrf2 exists in the cytoplasm, binds to Keap1, and is rapidly degraded via the proteasome pathway. However, upon cellular injury, Nrf2 dissociates from Keap1 and translocates to the nucleus, where it activates antioxidant response element-driven genes that mitigate oxidative damage [57]. This protective mechanism is particularly important in I/R injury, as Nrf2-knockout mice are shown to have increased I/R susceptibility, accelerated heart failure progression, and elevated mortality. Conversely, enhanced Nrf2 expression confers significant protection against oxidative injury [56]. Additionally, the activation of Nrf2 is essential for maintaining intestinal health since it not only regulates the production of tight junction proteins but also prevents the apoptosis of epithelial cells, controls the excessive proliferation of intestinal stem cells, and maintains a stable internal environment in the intestine [58]. In a variety of disease models, the activation of the Nrf2 has been found to effectively mitigate the damage to the intestinal barrier [[58], [59], [60]]. Nrf2 regulates the activity of HO-1, which is a class of antioxidant enzymes having a key role in the body's anti-inflammatory processes [61]. The Nrf2/HO-1 signaling pathway upregulates antioxidant-related genes and reduces inflammatory responses and oxidative damage in the body [33]. The current findings show that Nrf2 and HO-1 levels were significantly elevated in mice after II/R injury, and IQ further enhanced these levels. However, the effects of IQ were abolished in the presence of an Nrf2 inhibitor (ML385) or upon Nrf2 knockdown. Thus, IQ inhibited II/R-induced oxidative stress via the Nrf2/HO-1 axis, to thus strengthen the intestinal barrier and mitigate the disruption to the intestinal flora. The potential mechanism of IQ likely involved the Nrf2/HO-1-mediated inhibition of the oxidative stress, the protection of the intestinal barrier, and the inhibition of the NLRP3-mediated inflammatory response.

This study investigated the effects of IQ on II/R injury using an *in vivo* model of intestinal ischemia and two *in vitro* intestinal epithelial cell lines, IEC-6 and Caco-2. The results demonstrate that IQ exerts a protective effect during II/R injury by suppressing inflammation and oxidative stress, restoring intestinal barrier integrity, and normalizing gut microbiota homeostasis. Mechanistically, these effects are mediated through Nrf2/HO-1 signaling pathway activation and NLRP3 inflammasome inhibition. Although the findings are promising, some limitations must be acknowledged. First, macrophage infiltration plays a crucial role in both the progression of inflammation and tissue repair during ischemia [62,63]. Future studies will focus on investigating macrophage polarization in the context of intestinal ischemia, as well as the long-term effects of IQ on macrophage function during ischemic injury. Furthermore, while the protective effects of IQ against II/R injury in the small intestine were demonstrated thoroughly, the potential influence of IQ on other parts of the gastrointestinal tract was not evaluated. In addition, the mice in the *in vivo* study were given IQ for a short period (4 days) by intraperitoneal injection only, and the results could not confirm the impacts of IQ administered in the longer term or using other modalities. Despite these drawbacks, the current findings still suggest that IQ could be an effective agent to reduce II/R injury.

## CRediT authorship contribution statement

**Hui Xu:** Writing – review & editing, Writing – original draft. **Tian-qi He:** Writing – original draft, Data curation. **Su-ying Chen:** Supervision. **Rui-rui Shi:** Supervision. **Jian Xu:** Supervision. **Yu-run Xing:** Supervision. **Dan Shi:** Supervision. **Yi-qin Liu:** Supervision. **Bo-sheng He:** Supervision. **Jin-hua Gu:** Writing – review & editing, Conceptualization.

## Ethics approval and consent to participate

The animal study was approved by the Institutional Animal Care and Use Committee of Nantong University (Approval No. 20221211007).

## Consent for publication

All authors approved publication of the final manuscript.

## Availability of data and materials

Additional data are included in the Supplementary Information.

## Funding

This work was supported by the 10.13039/100017962Scientific Research Project of Health Commission of Jiangsu Province (MQ2024057), 10.13039/501100018557Science and Technology Project of Nantong City (JC2024028 and JC2024106), Nantong University Special Research Fund for Clinical Medicine (2024JY045), and the 10.13039/501100001809National Natural Science Foundation of China (81870941).

## Declaration of competing interest

The authors declare that they have no known competing financial interests or personal relationships that could have appeared to influence the work reported in this paper.

## Data Availability

Data will be made available on request.
