# Supplementary material for: Isoquercitrin mitigates intestinal ischemia-reperfusion injury by regulating intestinal flora and inhibiting NLRP3 inflammasome activation
Source: Redox Biol. 2025 Aug 5;86:103803. doi: 10.1016/j.redox.2025.103803 (PMC12357270; doi:10.1016/j.redox.2025.103803)

Fig3A

ZO-1 230 KDa

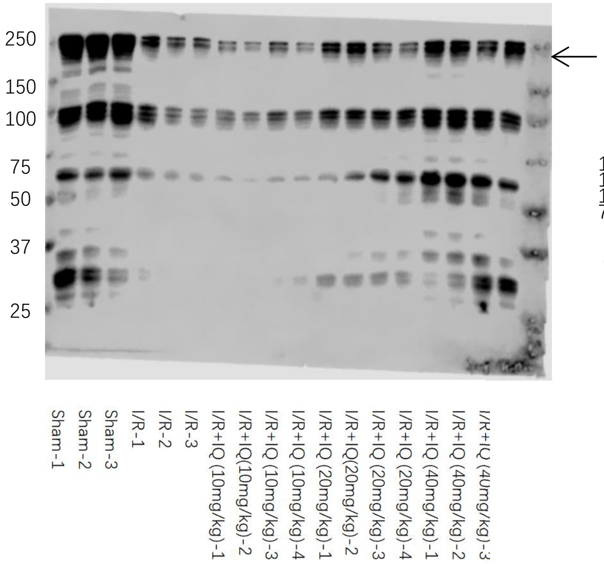

GAPDH 36KDa

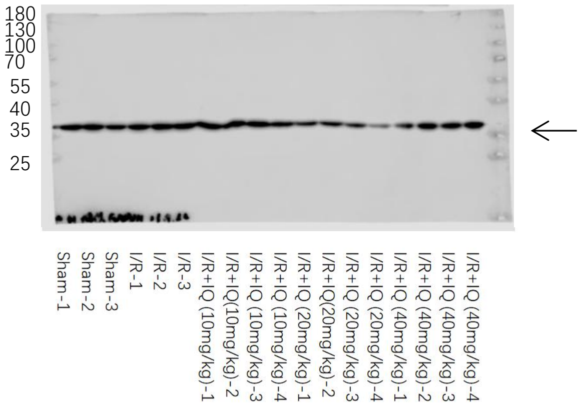

Fig3C

ZO-1 230KDa

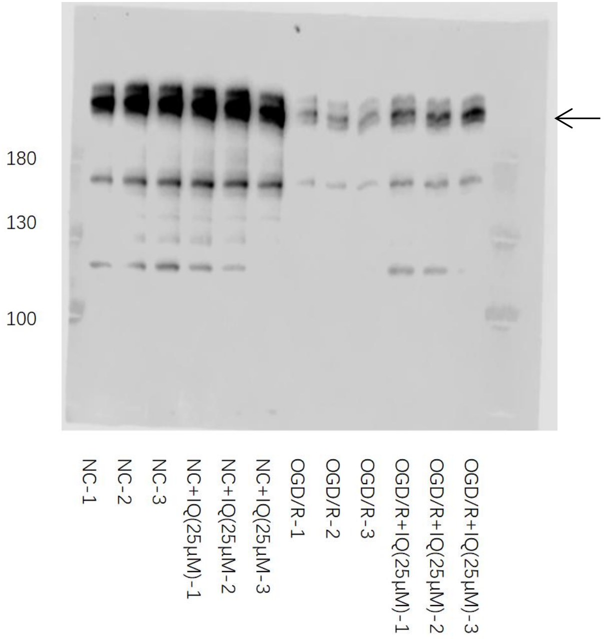

GAPDH 36KDa

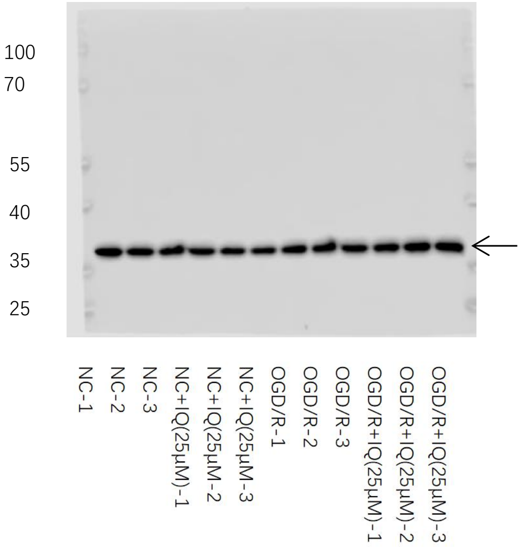

Fig3E

ZO-1 230KDa

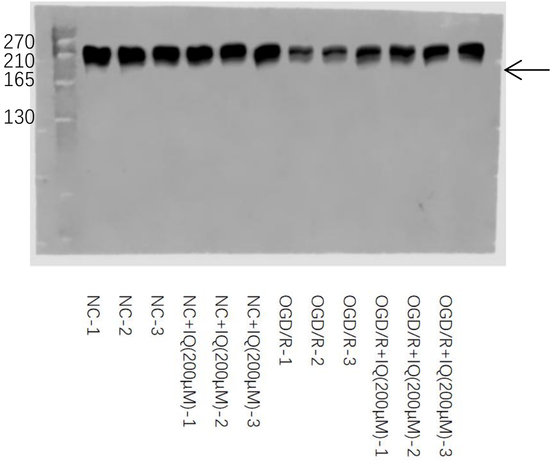

GAPDH 36KDa

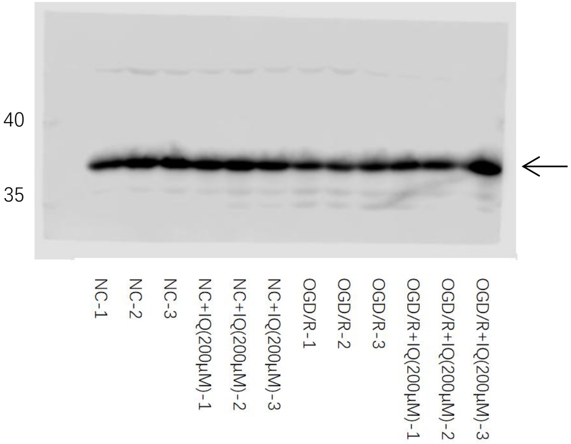

Fig6A

IL-1β 31KDa

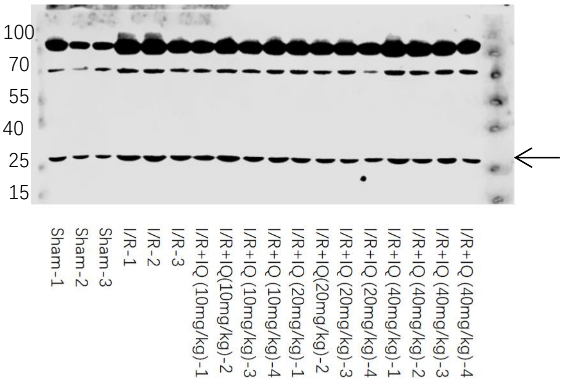

NLRP3 110KDa

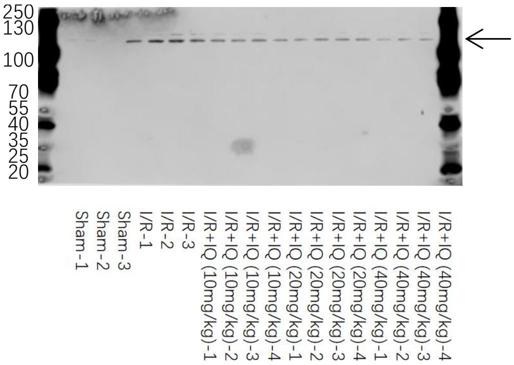

Caspase 1 45KDa

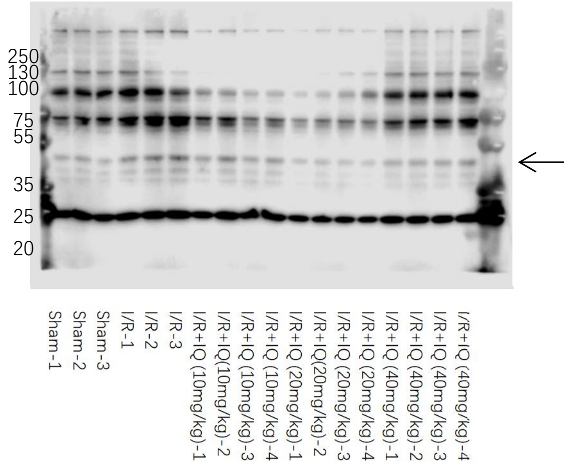

ASC 22KDa

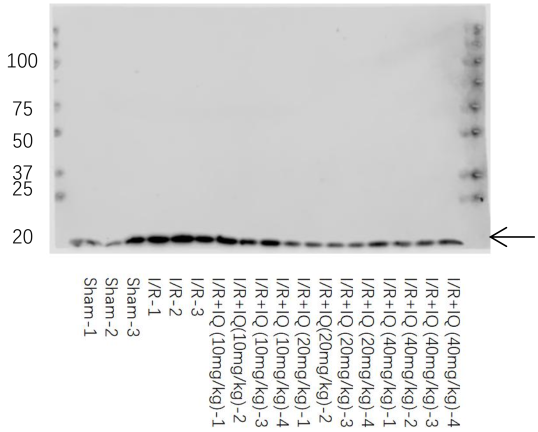

GAPDH 36KDa

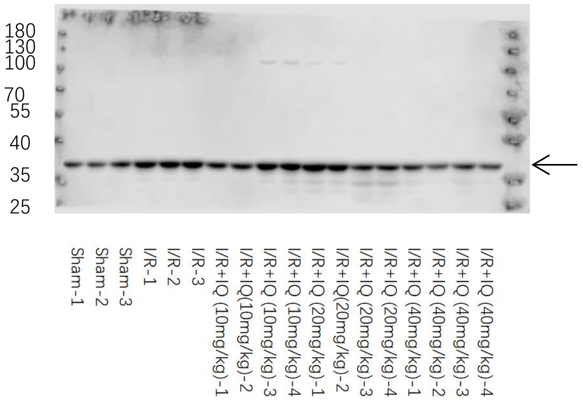

Fig6B

IL-1β 31KDa

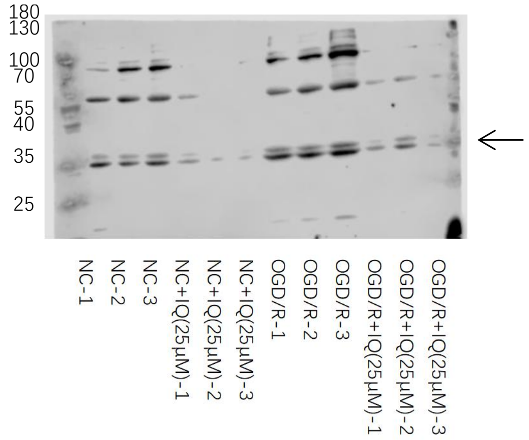

NLRP3 110KDa

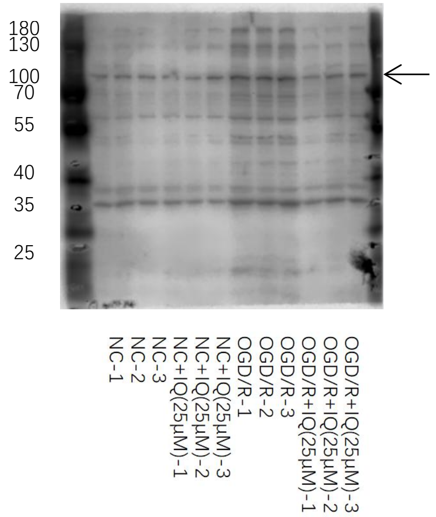

Caspase 1 45KDa

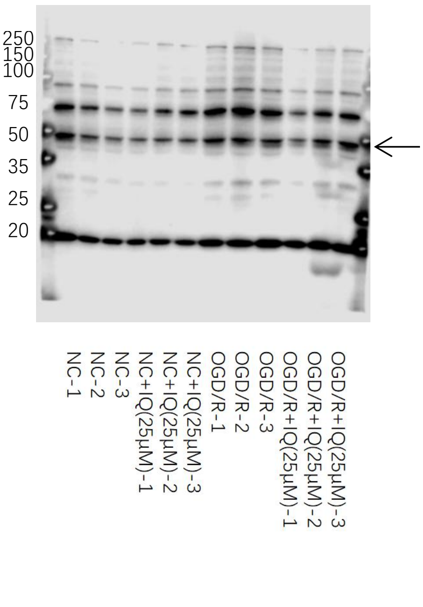

ASC 22KDa

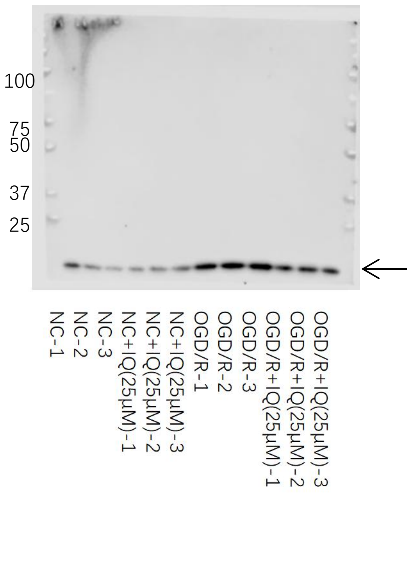

GAPDH 36KDa

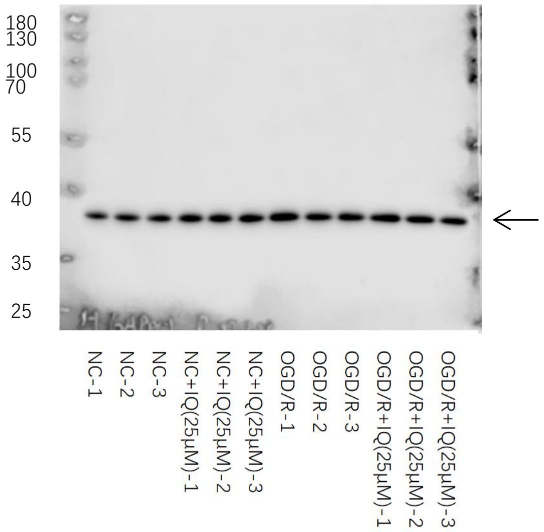

Fig10A

Nrf2 (Nuclear) 110 kDa

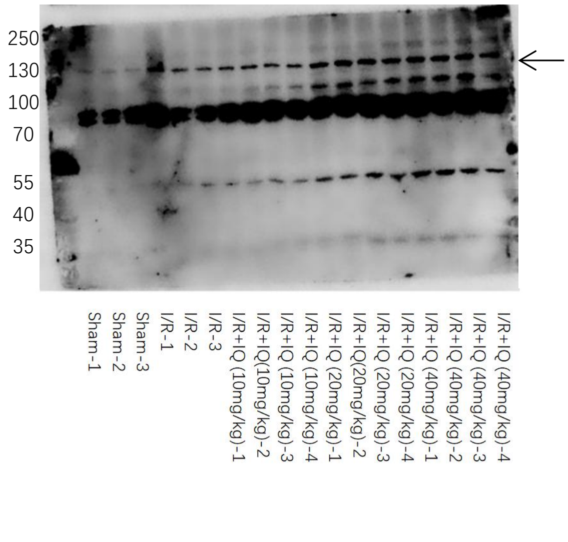

Lamin B1 (Nuclear) 66 kDa

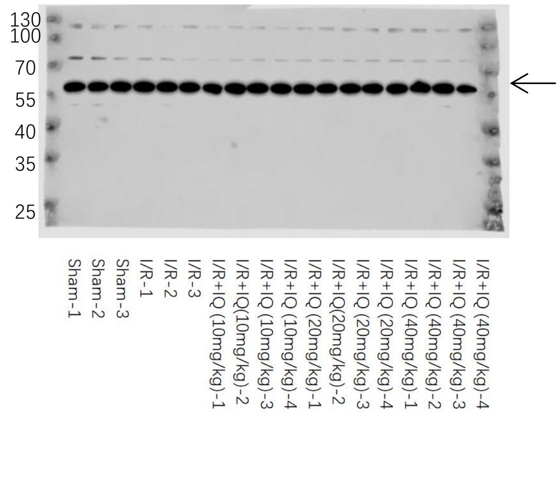

Nrf2 (Cytosolic) 110 kDa

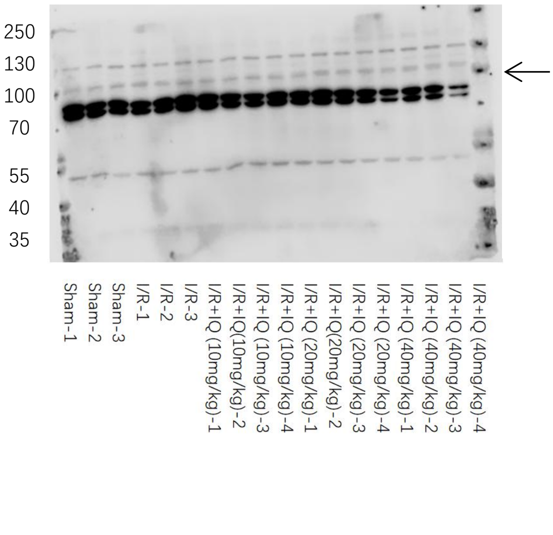

GAPDH (Cytosolic) 36 kDa

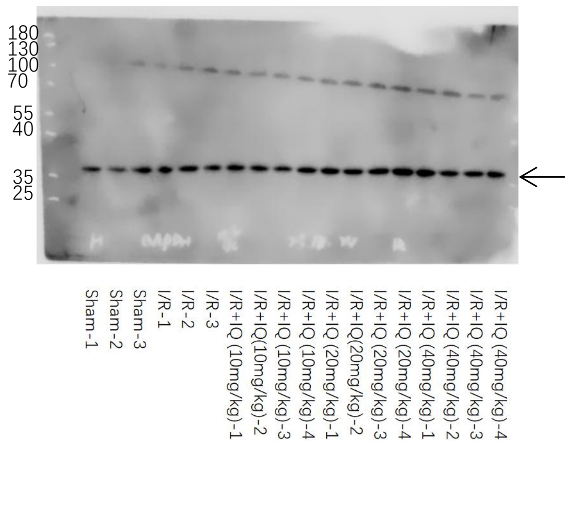

GAPDH (Nuclear) 36 kDa

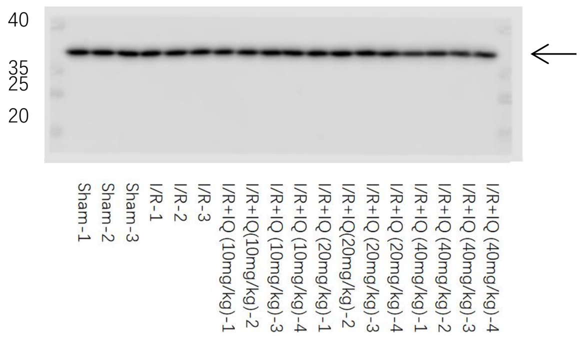

Nrf2 (Nuclear) 110 kDa

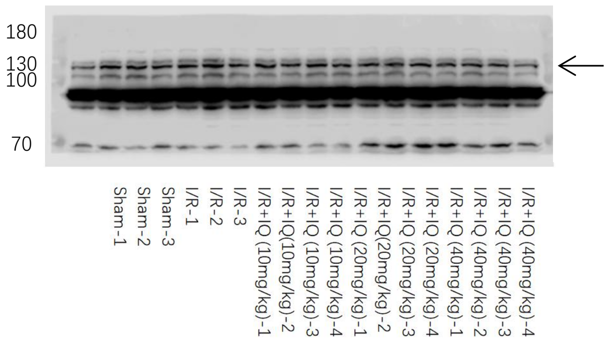

Fig10E

Nrf2 (Nuclear) 110 kDa

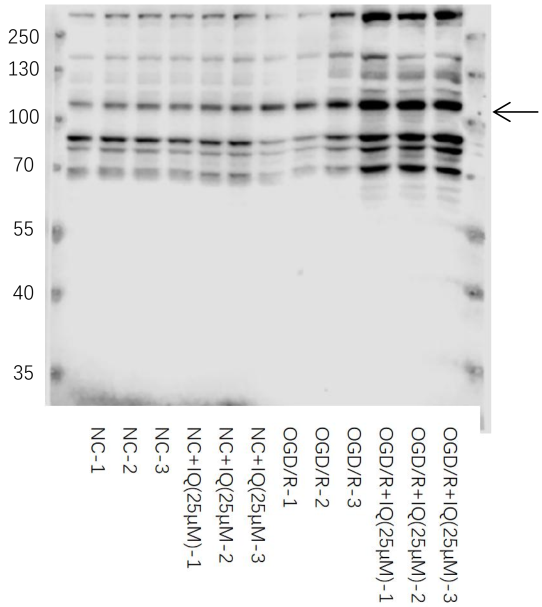

Lamin B1 (Nuclear) 66 kDa

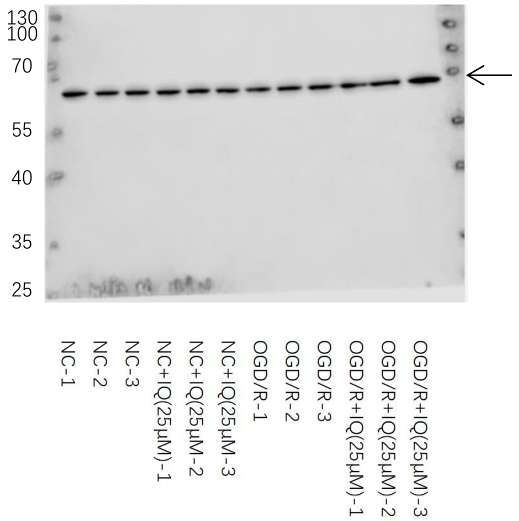

Nrf2 (Cytosolic) 110 kDa

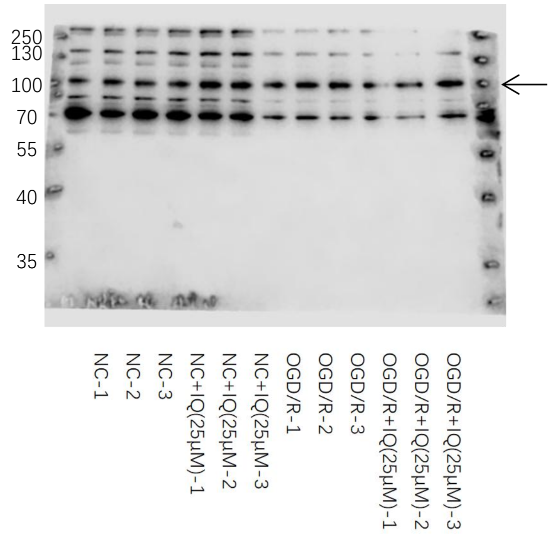

GAPDH (Cytosolic) 36 kDa

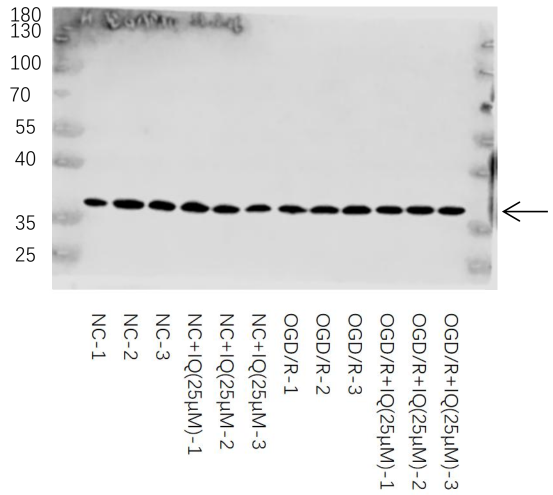

Nrf2 (total) 110 kDa

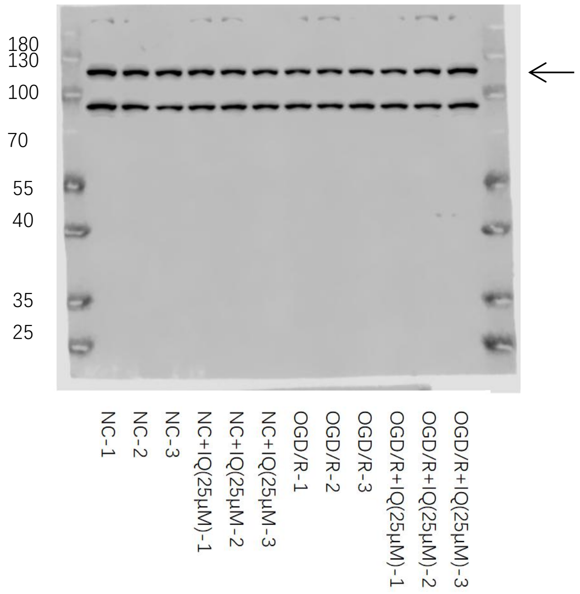

GAPDH (toatl) 36 kDa

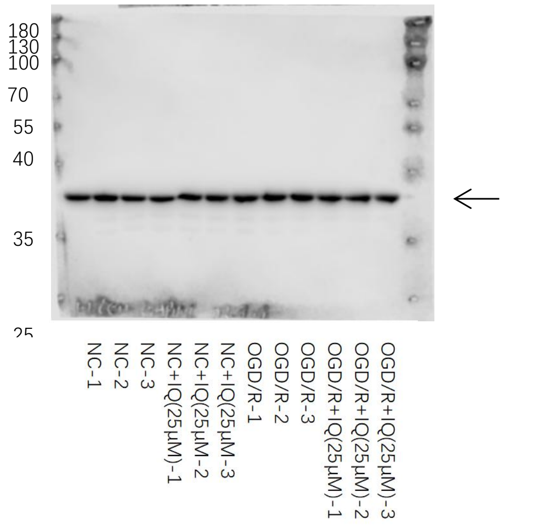

Fig10 G GAPDH (Cytosolic) 36 kDa

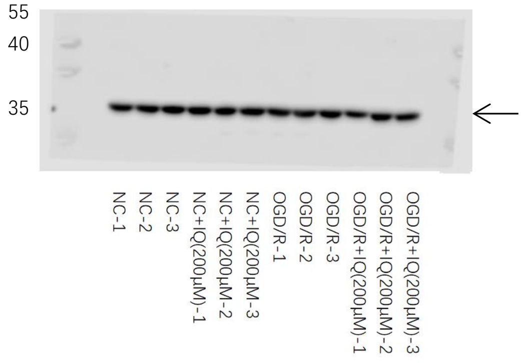

Fig10 G Nrf2 (Cytosolic) 110 kDa

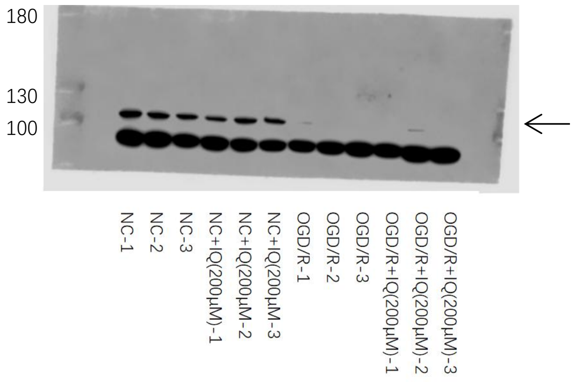

Fig10 G Nrf2 (Nuclear) 110 kDa

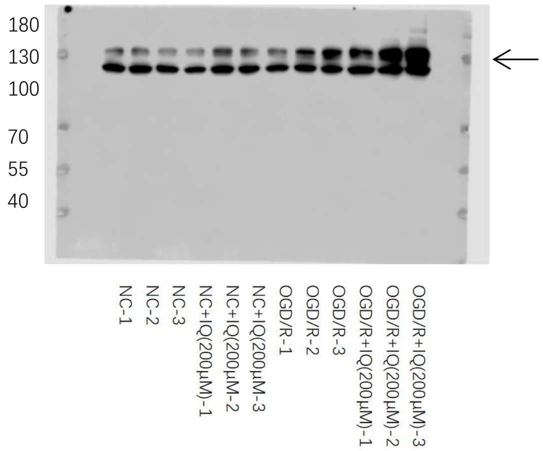

Lamin B1 (Nuclear) 66 kDa

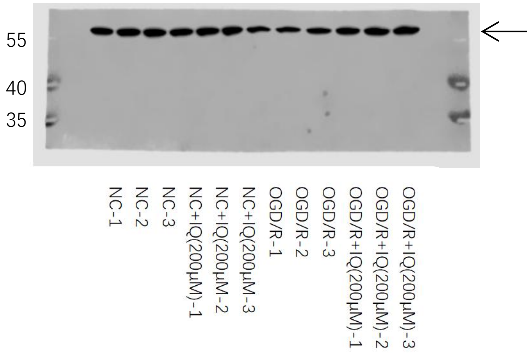

Fig10 G GAPDH (total) 36 kDa

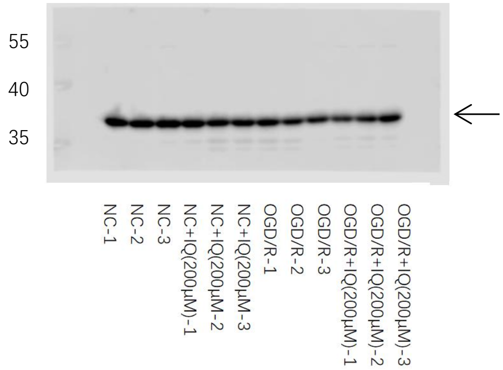

Fig10 G Nrf2 (total) 110 kDa

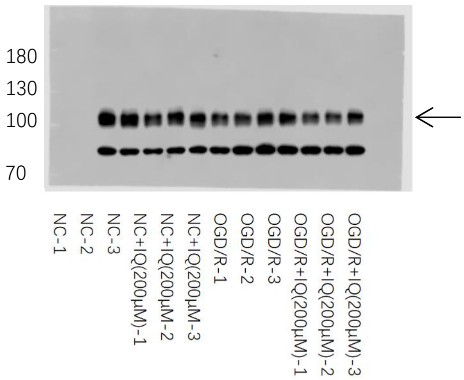

Fig11G

HO-1 33KDa

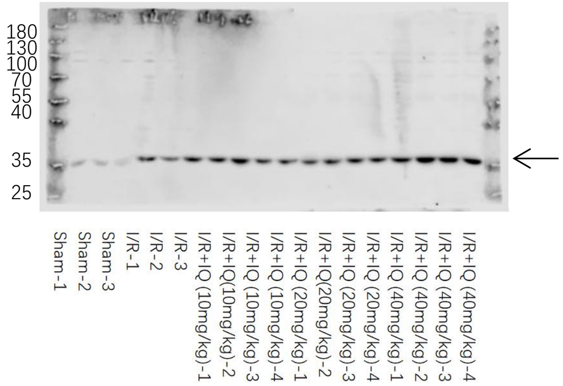

GAPDH 36KDa

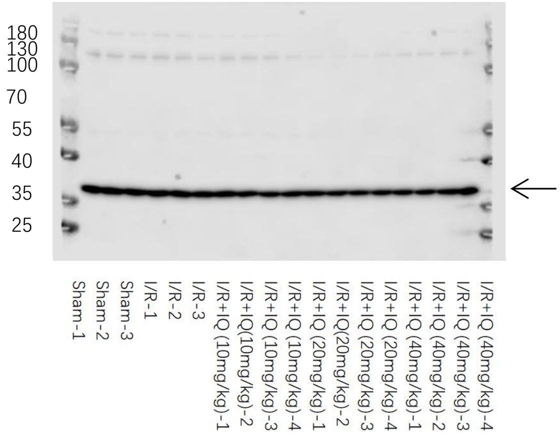

Fig11C

HO-1 33KDa

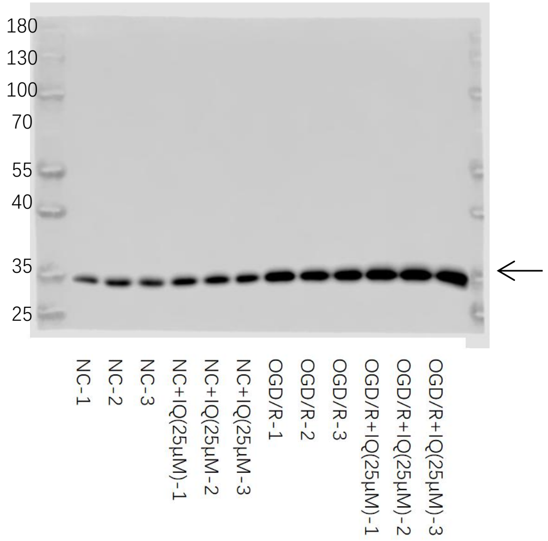

GAPDH 36KDa

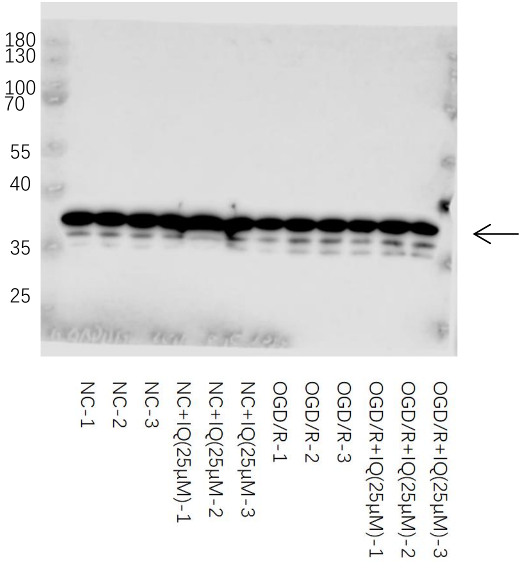

Fig11E

HO-1 33KDa

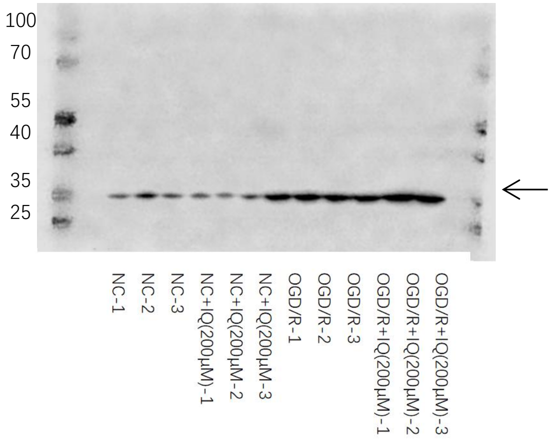

GAPDH 36KDa

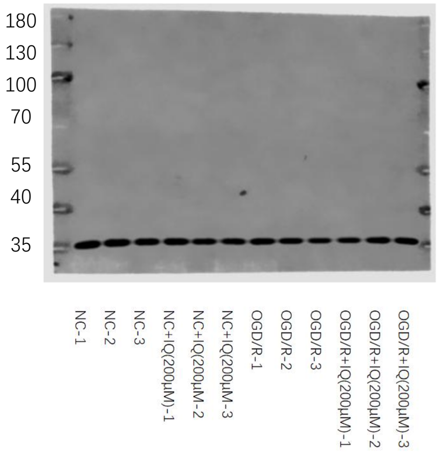

Fig12C

ZO-1 230KDa

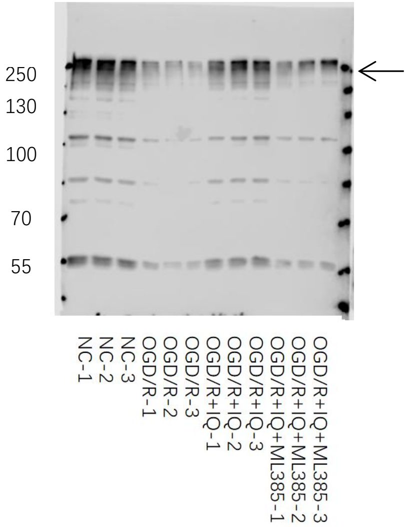

HO-1 33KDa

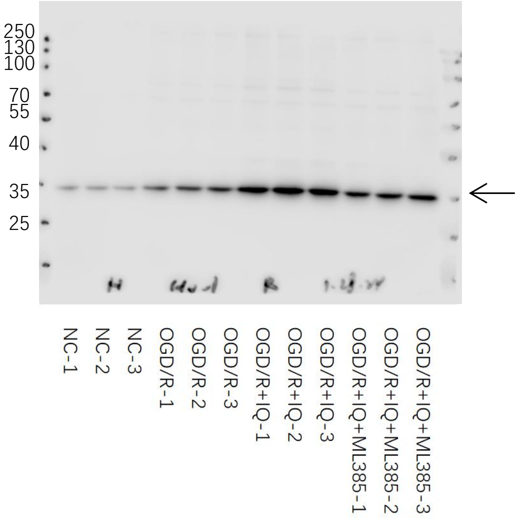

Nrf2 110KDa

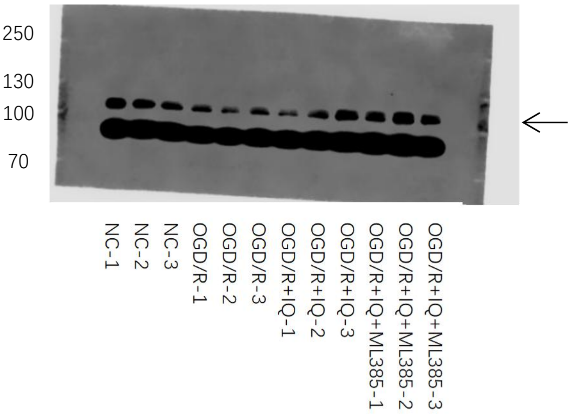

GAPDH 36KDa

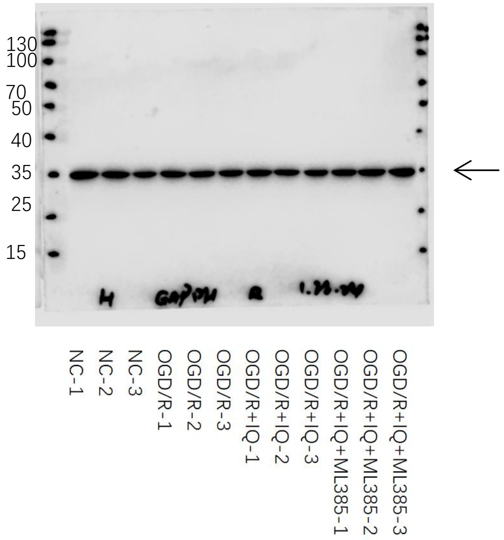

Fig12G Nrf2 (Nuclear) 110 kDa

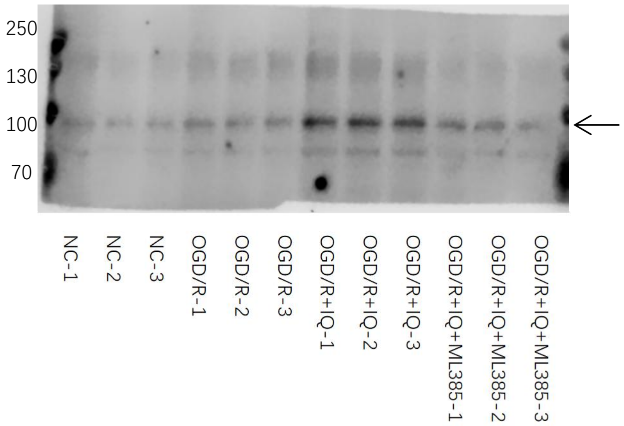

Lamin B1 (Nuclear) 66 kDa

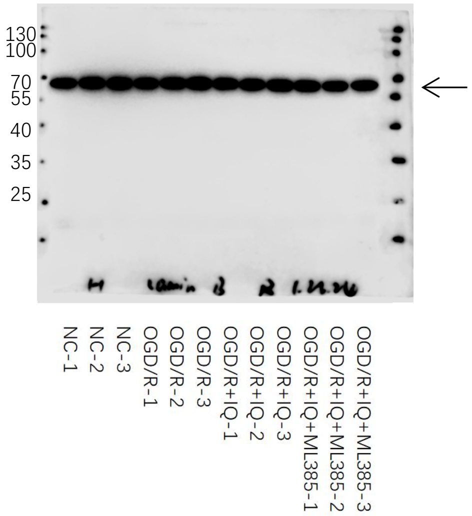

Fig12G

Nrf2 (Cytosolic) 110 kDa

GAPDH (Cytosolic) 36 kDa

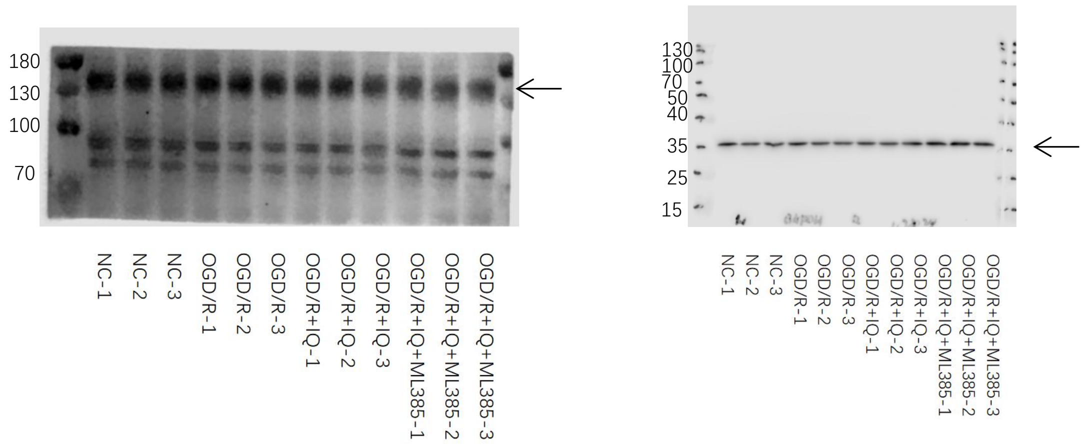

Fig13A

Nrf2 110 kDa

GAPDH 36 kDa

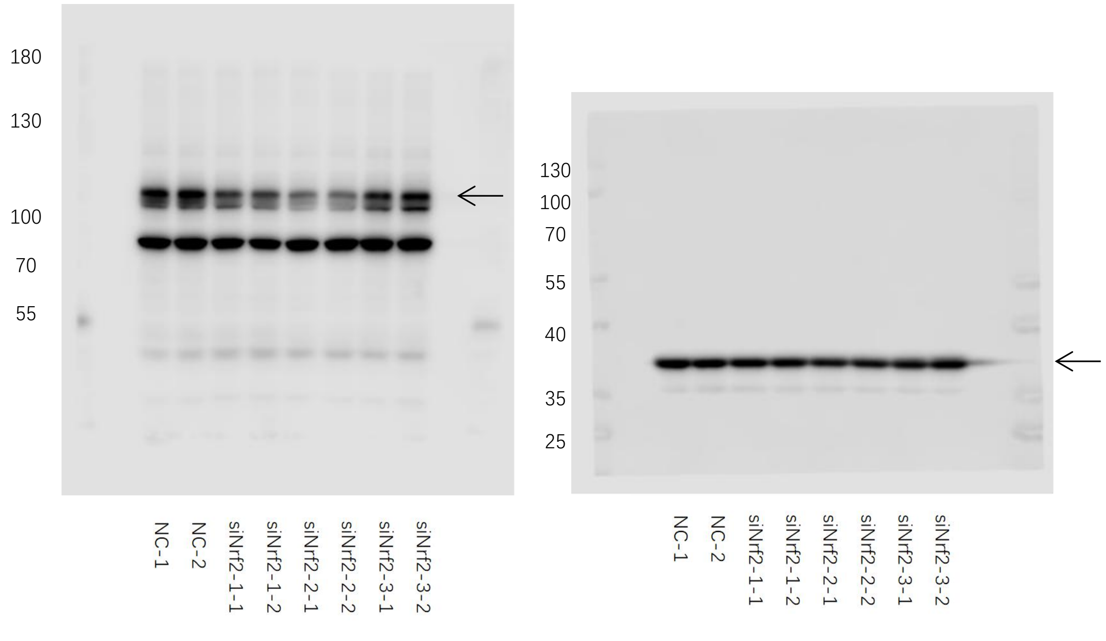

Fig13D

Nrf2 (Cytosolic) 110 kDa

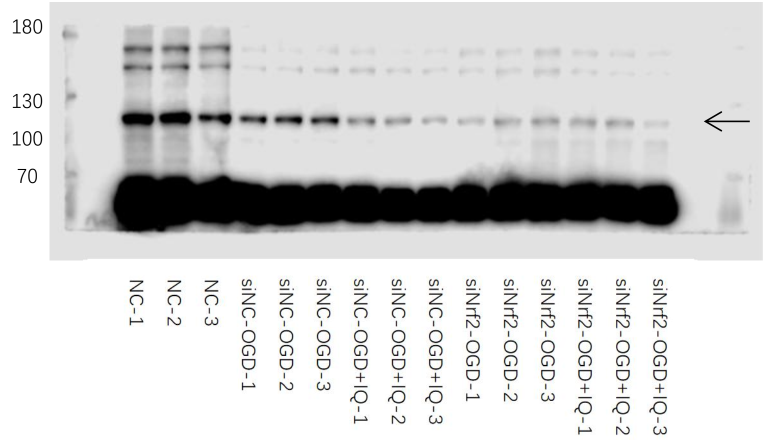

GAPDH (Cytosolic) 36 kDa

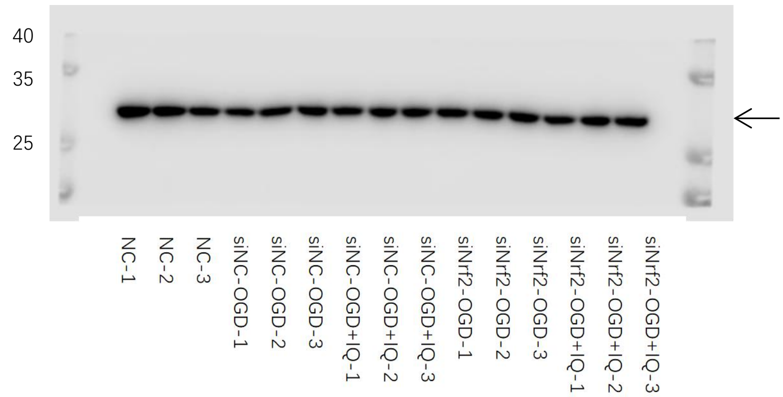

Fig13D Nrf2 (Nuclear) 110 kDa

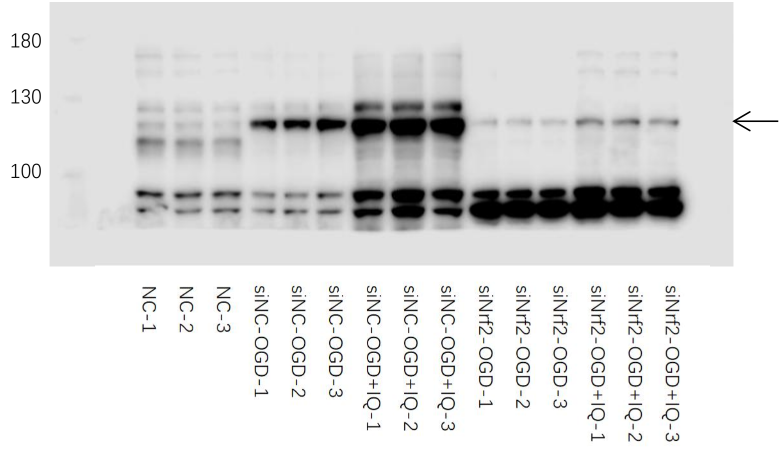

Lamin B1 (Nuclear) 66 kDa

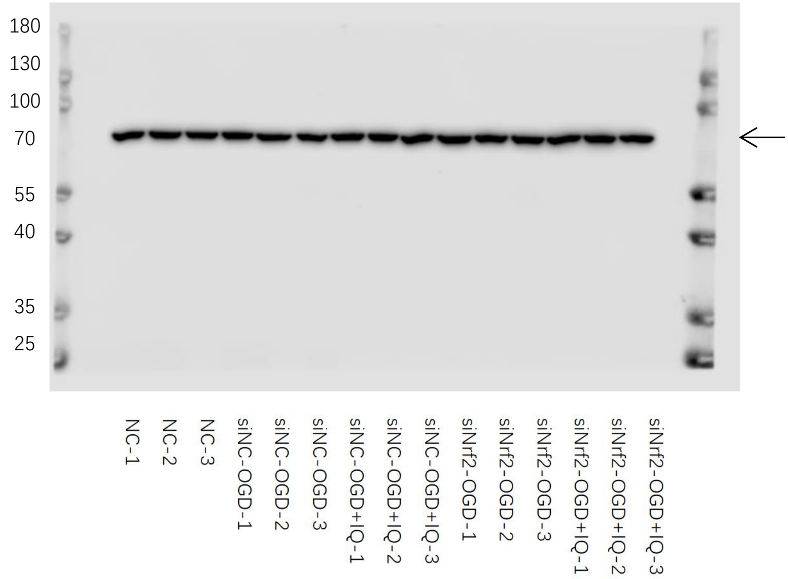

Nrf2 (total) 110 kDa

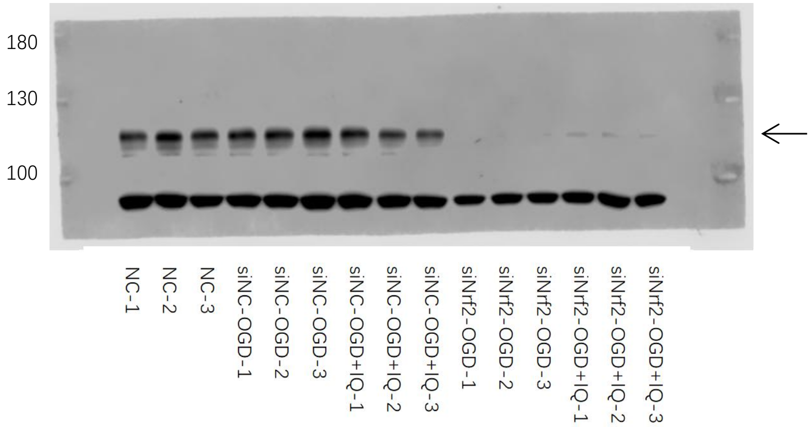

HO-1 33KDa

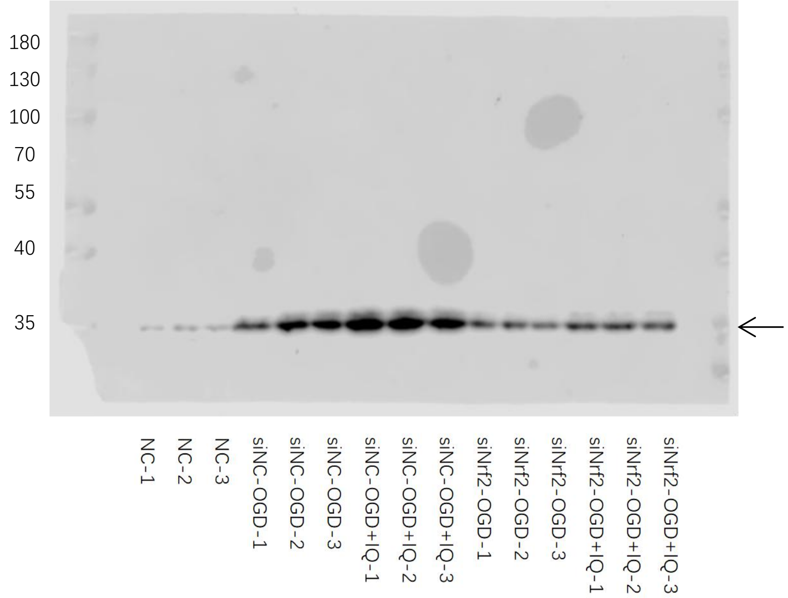

ZO-1 230 kDa

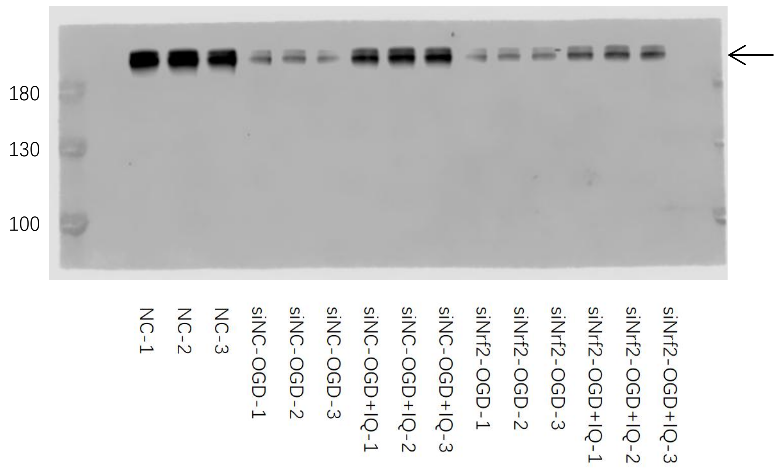

GAPDH 36 kDa

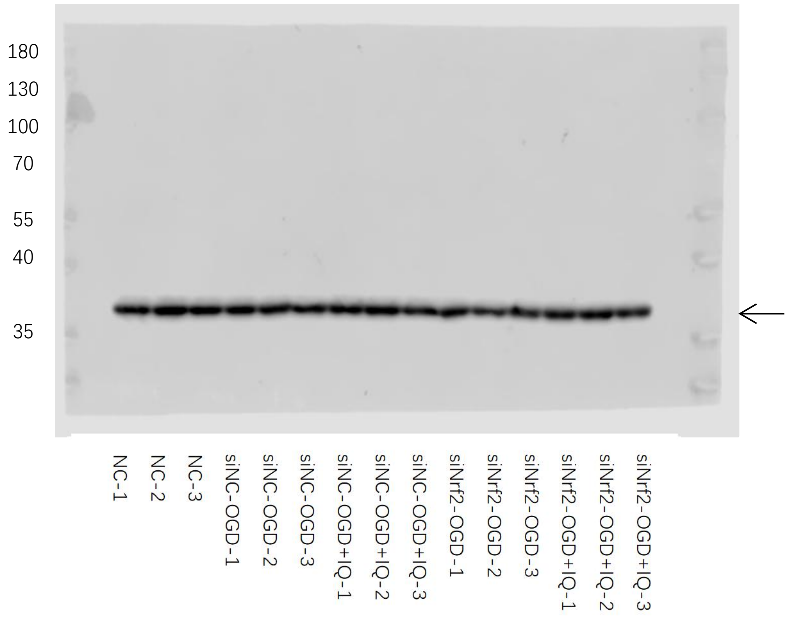

Supplement: Multimedia component 1 [file mmc1.pdf]
